# Development of a physical activity counseling intervention for people with chronic respiratory disease based on the health action process approach

**DOI:** 10.1186/s40814-023-01397-w

**Published:** 2023-10-12

**Authors:** Rachel S. Tappan, Jennifer R. Ettinger, Delaney Mahon, Sarah E. Mroz, Walter Hall, Estelle Maajid, Chelsea Stratton, Denise Trotter Zynda, David E. Conroy, Margaret Danilovich

**Affiliations:** 1 Department of Physical Therapy and Human Movement Sciences, Feinberg School of Medicine, Northwestern University, Chicago, USA; 2 Department of Medicine (Pulmonary and Critical Care), Feinberg School of Medicine, Northwestern University, 645 N. Michigan Avenue, Suite 1100, Chicago, Illinois 60611 USA; 3 Present Address: 933 Garden Lane, Homewood, Illinois 60430 USA; 4 Present Address: 536 East 32nd Street Unit E, Chicago, Illinois 60616 USA; 5 Department of Physical Therapy, Marquette University, Schroeder Complex, Room 346, P.O. Box 1881, Milwaukee, Wisconsin 53210 USA; 6 Present Address: 6057 N. Lincoln Avenue, #204, Chicago, Illinois 60659 USA; 7 Department of Kinesiology, Human Development & Family Studies and Public Health Sciences, The Pennsylvania State University, 268U Recreation Building, University Park, Pennsylvania 16802 USA; 8 Leonard Schanfield Research Institute, CJE SeniorLife, 3003 W. Touhy Avenue, Chicago, Illinois 60645 USA; 9 Feinberg School of Medicine, Northwestern University, Chicago, Illinois USA

**Keywords:** Chronic respiratory disease, Physical activity, Exercise, Counseling, Pulmonary rehabilitation, Behavior modification

## Abstract

**Background:**

Physical activity (PA) counseling holds promise for increasing PA levels in people with chronic respiratory disease, though little long-term change has been shown to date. Here, we describe the development of a Health Action Process Approach-based PA counseling intervention that aims to promote PA and exercise in people with chronic respiratory disease who are enrolled in pulmonary rehabilitation.

**Methods:**

To collaborate in defining and refining the intervention, we convened a varied team of authors that included a panel of five stakeholder partners: three patients, one clinician, and one health behavior change researcher. We completed three steps in the intervention development process: (1) initial intervention creation, (2) iterative intervention refinement, and (3) assessment of intervention acceptability. In step 1, we created an initial draft of the PA counseling intervention based on the HAPA theoretical framework, previous evidence in people with chronic respiratory disease, and clinical experience. In step 2, we used qualitative methods of focus groups and interviews to further develop and refine the intervention. Fifteen meetings occurred with the five-member stakeholder partner panel (six focus groups with the three patient partners, four interviews with the clinician partner, and five interviews with the researcher partner) over 5 months to systematically elicit input and incorporate it into the intervention. In step 3, we measured the intervention acceptability using five-point Likert scale ratings.

**Results:**

Intervention materials included the eligibility screen, participant workbook, and leader guide. We identified key themes in the input from the stakeholder partners and incorporated this input into the intervention content and methods. Ratings of the intervention by the stakeholder partners (*n*=5) were high with mean ratings ranging 4.0–5.0 on a five-point scale.

**Conclusions:**

This development process successfully engaged an intervention development team with diverse perspectives and resulted in a PA counseling intervention for people with chronic respiratory disease. The intervention’s strong theoretical underpinning, person-centeredness, and the contributions from varied perspectives during intervention development position it well for future evaluations of feasibility, efficacy, and effectiveness.

**Supplementary Information:**

The online version contains supplementary material available at 10.1186/s40814-023-01397-w.

## Key messages regarding feasibility 

- What uncertainties existed regarding the feasibility?
  - ◦ Prior studies have not identified an effective intervention for long-term improvement in physical activity in people with chronic respiratory disease. A feasible, acceptable, and effective intervention is needed.
- What are the key feasibility findings?
  - ◦ In this intervention development study, we incorporated patient, clinician, and researcher perspectives in order to increase the likelihood that the resulting intervention would be acceptable and feasible for implementation in a research study and in the clinic. Some example areas where this input resulted in refinement of the intervention include session timing, participant group characteristics, and format and content of the eligibility screen, participant workbook, and leader guide.
- What are the implications of the feasibility findings for the design of the main study?
  - ◦ The intervention development process was designed to increase the likelihood of the intervention being feasible in a research and clinical context. The feasibility of the intervention remains to be tested in future studies.

## Background

People with chronic respiratory disease exhibit low physical activity (PA) levels and reduced exercise capacity [1–3], both of which are associated with poor overall health outcomes [2, 4–6] and lower quality of life [7]. While interventions that lead to short-term increases in exercise capacity in people with chronic respiratory disease are well-established, interventions that lead to long-term improvement in PA and exercise capacity remain elusive. In this paper, we describe the development of a new intervention that aims to close this gap.

Pulmonary rehabilitation, which includes exercise training, education, and behavior change interventions [2, 8], leads to improvements in exercise capacity, muscle function, dyspnea, cardiovascular function, symptom burden, and quality of life [2, 9]. Unfortunately, these health gains often diminish within 12 months [10]. The limited long-term effects of pulmonary rehabilitation may be due, in part, to low exercise adherence after program completion [2] and the limited impact of pulmonary rehabilitation on overall PA [11]. Continued supervised exercise can attenuate this decline [12, 13], but access to ongoing supervised exercise is often limited. Therefore, it is critical to develop new interventions that will maximize unsupervised PA after pulmonary rehabilitation completion, including both daily PA and exercise. 

PA counseling is one intervention with promise for increasing unsupervised PA levels in people with chronic respiratory disease. In PA counseling, participants engage in counseling through a person-centered approach to implement motivational and volitional strategies to increase and maintain PA behavior [11, 14]. Some PA counseling interventions delivered in combination with pulmonary rehabilitation do bring about small to moderate increases in short-term PA, but little long-term change [11, 15, 16]. We hypothesize that effectiveness of PA counseling interventions for people with chronic respiratory disease can be further improved by incorporating more components of health behavior change. For instance, most previously developed interventions do not account for participants’ varying intention to increase PA behaviors, participants’ preferences regarding strategies and activities, participants’ struggles to translate their intentions into action (i.e., the intention-behavior gap), or planning for lapses in PA behavior [14]. In addition, we hypothesize that people who are intending to change their PA behaviors may benefit from different interventions than people who do not have this intention. For instance, goal setting and activity planning may be counterproductive for a person who has no intention to exercise in the first place. The Health Action Process Approach (HAPA) is a contemporary theoretical framework for health behavior change that incorporates these considerations [17]. Thus, we propose that PA counseling for people with chronic respiratory disease could be enriched by using HAPA as the theoretical underpinning for intervention design. 

The aim of this project was to develop a PA counseling intervention that is guided by HAPA and incorporates stakeholder input to promote PA and exercise behavior in people with chronic respiratory disease who intend to increase their PA levels. In this paper, we describe phase 1 of the development process for a theory-based behavior change intervention for people who are enrolled in an outpatient pulmonary rehabilitation program and who intend to increase their PA level. This process focuses on defining and refining theory-based intervention content and methods based on stakeholder feedback [18]. 

## Methods

### Design and rationale

We used an action research-based approach [19] with qualitative methods of focus groups and interviews to develop a PA counseling intervention with patient, clinician, and researcher partners as members of the intervention development team. We purposefully included team members with these varied perspectives to increase the likelihood of future feasibility, acceptability, and effectiveness of the intervention, which will be investigated in future trials. For the purposes of this paper, “stakeholder partner” refers to these patient, clinician, and researcher members of the co-author team. “Participant” refers to people who will participate in the intervention in future research or clinical activities. 

We completed three steps in the intervention development process: (1) initial intervention creation by a subset of the intervention development team, (2) iterative intervention refinement by a subset of the intervention development team based on feedback from remaining members (the stakeholder partners), and (3) assessment of the final intervention by the stakeholder partners to ensure that the changes from step 2 resulted in an acceptable product overall. This three-step process provided an efficient and effective means for all intervention development team members’ input to be included. 

### Intervention development team

The intervention development team included (1) an academic faculty member with expertise in pulmonary rehabilitation (RST), (2) an academic faculty member with expertise in health behavior change in older adults (MD), (3) three physical therapist trainees (JRE, DM, SEM), and (4) a five-member stakeholder partner panel consisting of patients (WH, EM, DTZ), a clinician (CS), and a researcher (DEC). The three patient partners provided a perspective representative of people with chronic respiratory disease who would be appropriate for future participation in the intervention. They have a history of chronic respiratory disease and have participated in at least 4 weeks of pulmonary rehabilitation in the past. The patient partner group included representation of diagnoses with both obstructive and restrictive impairments. The clinician partner is a physical therapist with experience and expertise in pulmonary rehabilitation who provided the perspective of a clinician who could implement the intervention in a clinical context. The researcher partner is a researcher with expertise in health behavior change who provided the perspective of a researcher who could implement the intervention in a research context. The patient partners were recruited through clinical contacts with local pulmonary rehabilitation programs. The researcher and clinician partners were recruited through professional networks with the other members of the authorship team. 

### Step 1: initial intervention creation

Four authors (RST, JRE, DM, SEM) wrote the initial draft of the standardized curriculum for this multi-session intervention implemented by a clinician leader with participants who intend to increase their physical activity levels and who are also participating in outpatient pulmonary rehabilitation. The goal of the intervention is to increase participants’ long-term PA through a focus on daily PA and exercise behavior that incorporates participant preferences, needs, and resources. 

**Fig. 1:**
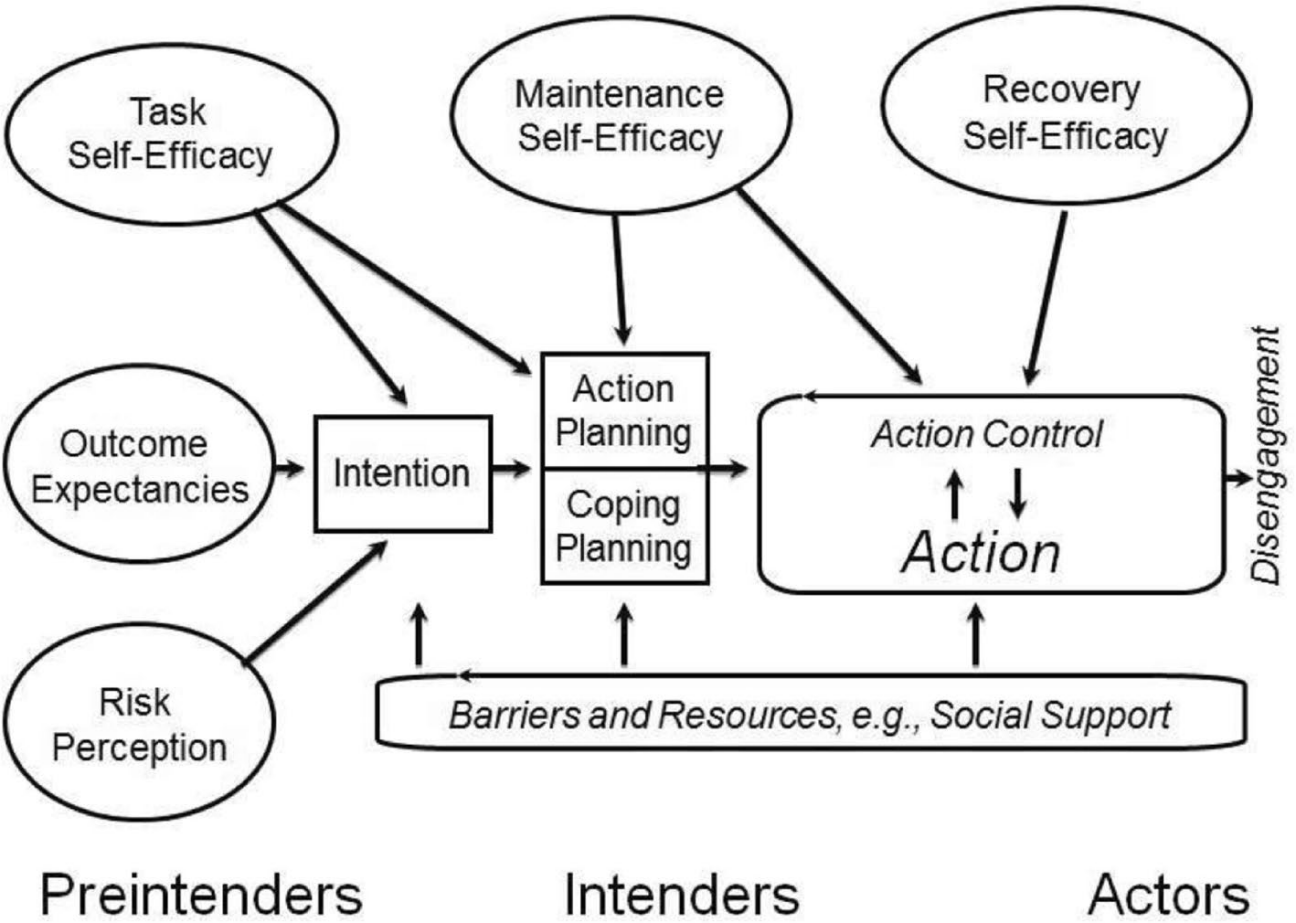
Health Action Process Approach. Schwarzer R. The Health Action Process Approach (HAPA), https://www.hapa-model.de/ (accessed August 11, 2022)

The initial intervention draft included four 60 to 90-min sessions provided in a group format, consistent with one author’s (RT) experience as a clinician providing the education component of traditional pulmonary rehabilitation. The Health Action Process Approach (HAPA) was systematically incorporated into the curriculum during session design by creating at least one session activity to address each component of the HAPA framework [17, 20]. We selected HAPA because it addresses all stages of health behavior change from forming intentions to turning those intentions into actions to maintaining the behavior over time. HAPA is a social-cognitive framework and includes six key constructs: (1) intention to change, (2) risk perception, (3) outcome expectancies, (4) self-efficacy (i.e., task, maintenance, and recovery self-efficacy), (5) planning (i.e., “action planning” to create a plan for the behavior and “coping planning” to create a plan for how to resume the behavior in the event of a relapse), and (6) action control (i.e., maintaining the behavior) [17] (Fig. 1). Risk perception, outcome expectancies, and task self-efficacy all influence a person’s intention to engage in the health behavior, whereas task and maintenance self-efficacy and planning help to turn intentions into actions. Maintenance and recovery self-efficacy influence action control to maintain the health behavior. The person’s barriers and facilitators for the health behavior influence the entire continuum of health behavior change.

Written materials associated with this intervention included an eligibility screen, a participant workbook, and a leader guide. According to HAPA, people with different levels of intention to engage in the target behavior benefit from different behavior change techniques [20]. Therefore, this PA counseling intervention was designed to be specific for people with chronic respiratory disease who *intend* to increase their PA, and the *eligibility screen* aimed to identify appropriate participants who have intention to change in the next 6 months. While the focus of this intervention is on participants who have identified an intention to increase PA, participants may have stronger or weaker intentions. Therefore, the first session of this intervention included a focus on strengthening intentions while the remaining sessions focused on the volitional phase of HAPA. The *participant workbook* reflected the session content and homework activities. The workbook format allowed participants to record their active learning, reflection, and planning activities. To optimize readability for people with a wide range of health literacy levels, the participant workbook text incorporated short sentences and words with few syllables when possible. The *leader guide* provided guidance for the methods, content, and guiding principles to maximize intervention fidelity. The participant workbook and the leader guide incorporated evidence-based behavior change techniques such as monitoring and goal setting, known to improve PA for those with chronic obstructive pulmonary disease [21]. Principles of motivational interviewing [22, 23] were incorporated into the workbook materials and leader guide because they provide evidence-based strategies for influencing health behavior change that are consistent with HAPA’s consideration for the participants’ intention to change. 

### Step 2: intervention refinement

Stakeholder partner meetings were held with interview and focus group methods to systematically elicit and incorporate stakeholder partners’ input in an iterative fashion. We held separate meetings for the patient partners, clinician partner, and the researcher partner to allow the discussion to focus on the partners’ different areas of expertise. Stakeholder partner meetings included discussion about the content and format of each intervention component as well as the content, format, and participant outcomes for the overall intervention. Each meeting covered one to three new intervention components (e.g., eligibility criteria, session 1, session 2) depending on time constraints, plus a review of all edits to the intervention resulting from previous stakeholder partner input. Patient partner meetings also included simulation of portions of the intervention to provide meaningful context for patient partner discussions and to evaluate session content, flow, and timeframes for session activities. Additional meetings were held until all components of the intervention and resulting edits were covered. Topic guides for each of these meetings included semi-structured questioning about the intervention components listed above as well as opportunities for open-ended feedback. Topic guides were adjusted by authors DM, JRE, RST, and SEM prior to each meeting to reflect progress made in previous meetings. All stakeholder partner meetings occurred via online video call (Zoom Video Communications, Inc., San Jose, CA) due to the COVID-19 pandemic and geographic distance between team members. Each meeting was recorded and transcribed. One author (DM, JRE, RST, or SEM) recorded field notes in each meeting, and these notes were edited and verified by the other three authors. 

### Step 3: intervention evaluation by stakeholder partners

After all stakeholder partner meetings were completed, we administered an online survey via Research Electronic Data Capture (REDCap) to investigate stakeholder partner assessment of the final version of the intervention. The survey included (1) seven questions about perceptions and acceptability of the intervention rated on a 5-point Likert scale, (2) ratings of the importance of nine potential outcome constructs rated on a 5-point Likert scale, and (3) open-ended question for additional feedback. Questions with ratings on a 5-point Likert scale also offered the option of rating “unsure.” 

### Data analysis

One author (RT) analyzed the field notes and meeting transcripts after each stakeholder partner meeting to identify patterns of comments or single comments that described intervention elements that were important to keep and where changes would lead to an improvement. The same author proposed edits to address these elements. Three authors (DM, JRE, SEM) reviewed and verified the analysis and proposed edits after each meeting. The edits were then reviewed with all stakeholder partners in subsequent meetings to confirm agreement. In this way, intervention refinement was an iterative process that incorporated member checking with the stakeholder partners. We used descriptive statistics to analyze Likert ratings from the online survey using Microsoft Excel. Answers to open-ended survey questions are reported verbatim. 

This clinical quality improvement project represents preliminary activities intended to create and refine an intervention that can be investigated in larger scale studies in the future. The Northwestern University Institutional Review Board reviewed this study and determined it to be “not human research” (STU00211027). 

## Results

During step 2, we held six 1-h meetings with the patient stakeholders, four 30- to 45-min meetings with the clinician stakeholder, and five 30-min meetings with the clinician stakeholder over 5 months. The stakeholder partners’ comments were categorized as targeting the intervention format, content, or outcome and as being related to the overall intervention, eligibility screen, participant workbook, or leader guide. Elements categorized as being related to format included the structure of the intervention materials as well as the intervention sessions (e.g., session timing, number of participants) and elements categorized as being related to content included the concepts and expression of those concepts in the intervention materials and sessions. Elements categorized as being related to outcome included areas where participants may find value and impact of this intervention and that should be included in future investigation of intervention effectiveness. 

The final version of this PA counseling intervention consists of five 60-min sessions delivered in a group setting over a 9-week duration and is outlined in Table 1. Intervention materials include eligibility screen (see Additional file 1), participant workbook (see Additional file 2), and leader guide (see Additional file 3). The participant workbook materials are at a seventh grade reading level according to the Flesch Kincaid Grade Level.

**Table 1:** Physical activity counseling intervention curriculum outline with sessions delivered over a 9-week duration (weeks 1, 2, 3, 5, and 9)

Session	Outline	HAPA components addressed
1 –pre-work	• Reflection on o PA and exercise definitions o Experience with exercise o Outcome expectancies and risk perceptions	• Outcome expectancies • Risk perception
1	• Introductions • Establish group guidelines	
	• Pre-work review: o PA and exercise definitions o Outcome expectancies and risk perception of PA/exercise	• Same as pre-work
	• Discussion of past successful PA/exercise experiences • Exploration of benefits and costs (i.e., pros and cons) of changing PA health behavior or remaining in current state • Reinforcement for increasing PA	• Task self-efficacy • Maintenance self-efficacy • Outcome expectancies
2 –pre-work	• PA and exercise idea generation	• Action planning
2	• Education about PA and exercise recommendations and benefits, including cardiovascular exercise, resistance training, and quantifying PA/exercise parameters	
	• PA/exercise planning for the next week with adjustment as needed to increase self-efficacy in task completion	• Action planning • Task self-efficacy • Maintenance self-efficacy
3 –pre-work	• Completion of one-week PA/exercise plan	• Action/action control
	• Goal setting for 1 and 2 weeks	• Outcome expectancies • Action planning
3	• Goal review from pre-work • Reflection on impact of goals on life	• Same as pre-work
	• Setting two-week PA/exercise plan	• Action planning
	• Education about physical, intrapersonal, and interpersonal resources • Resource identification	• Resources
	• Self-efficacy review and planning to increase self-efficacy for PA/exercise plan	• Task self-eefficacy • Maintenance self-efficacy
4 –pre-work	• Reflection on plan implementation • Revision of goals from session 3 as needed	• Outcome expectancies • Action planning
4	• Identification of barriers encountered • Idea generation for overcoming barriers • Identification of resources used	• Barriers • Coping planning • Resources
	• Goal update from pre-work and action planning for next month	• Same as pre-work • Action planning
	• Exploration of self-efficacy in plan maintenance in the face of barriers and identification of ways to increase self-efficacy	• Task self-efficacy • Maintenance self-efficacy
	• Coping strategy generation for anticipated future barriers • Reflection on recovery self-efficacy and identification of helpful coping strategies	• Barriers • Coping plan • Recovery self-efficacy
5 – pre-work	• Continued PA/exercise plan implementation • Reflection on plan implementation consistency, barriers, resources, lapses, and coping strategies	• Outcome expectancies • Action/action control • Barriers • Resources • Coping plan • Recovery self-efficacy
5	• PA/exercise plan implementation review and related reflections from pre-work • Education and planning for exercise progression • Goal progression	• Same as pre-work • Action/action control • Maintenance self-efficacy

### Stakeholder partner input—interviews and focus groups

#### Overall intervention

All stakeholder partners felt that it was important to maximize self-reflection (content) and group interaction (format). Patient partners suggested restructuring sessions so that some content from each session was completed in a homework format (format) to give more time for self-reflection and to maximize group interaction time during each session. The clinician partner stated that the feasibility of the intervention was dependent on session timing and group composition to meet clinic and participant needs (format). Furthermore, the duration of each session should take into consideration that this intervention would be delivered in-person on the same day as pulmonary rehabilitation exercise sessions and therefore would require a longer per-day time commitment on the part of the participants and the clinician providers (format). Therefore, the following changes in intervention format were made: (1) reduced PA counseling session duration to 60 min or less to meet typical clinic staffing and participant transportation needs, (2) group size limited to a maximum of six participants to allow for optimal participant interaction, and (3) intervention sessions start by the midway point of a pulmonary rehabilitation program and extend beyond the end of the pulmonary rehabilitation program. Collectively, the stakeholder partners also provided the following suggestions for intervention outcomes that should be incorporated into future study (outcome): PA levels (self-reported and device-measured), exercise capacity, dyspnea, self-efficacy and motivation for PA, satisfaction with the intervention, adherence to intervention processes, and quality of life. 

#### Eligibility screen

The patient partners felt that it was important that participants believe that exercise will be helpful for this intervention to be effective, which supported the use of the eligibility screen to identify and select participants who intend to increase their PA (content). The clinician partner stated that it was key to identify appropriate participants early in pulmonary rehabilitation to facilitate enrollment in the intervention prior to discharge from pulmonary rehabilitation (format). Further, the researcher partner suggested including additional information in the eligibility screen to more accurately separate participants by level of intention and to better understand their exercise history (content). With this information, the eligibility screen was modified to (1) include guidance for timing of the screening and (2) identify people who intend to *start* engaging in structured PA or exercise and people who are already engaging in PA or exercise and intend to *increase* their PA or exercise in order to target participants most likely to benefit from the intervention. 

#### Participant workbook

All stakeholder partners agreed that the intervention should provide detailed education and resources for PA and exercise to augment the education and training provided during pulmonary rehabilitation (content). Specific suggestions included adding detailed information about exercise (e.g., dyspnea and rating of perceived exertion scales, definitions of exercise intensity, benefits and risks of exercise, exercise log examples, and local community resources for exercise) so that participants could effectively develop their individual plan and goals for PA. In order to encourage participation, the stakeholder partners identified the need to focus on the positive aspects of PA and exercise and to avoid highlighting negative experiences or expectations (content). To address these elements, the intervention was modified to increase focus on the participants’ reasons for wanting to increase their PA or exercise and limiting discussion of barriers to those that the participants encountered or that they were likely to encounter. Changes in the discussion of PA resources were to (1) focus on examples of modifiable traits rather than non-modifiable traits and (2) structure discussion so participants would be less likely to feel demotivated if they did not identify with a particular resource. 

#### Leader guide

Researcher and clinician partners believed the leader guide should focus on skills and principles rather than specific scripts to read (content). Examples suggested included a description of guiding principles such as focusing on collaborative goal setting, using active listening skills, and incorporating motivational interviewing principles. A list of HAPA elements and behavior change techniques [24] was also included in the leader guide description of each session. Other leader guide suggestions included orienting participants to steps previously completed in each session to provide context for each discussion (content), reminding participants to complete homework to maximize follow-through (content), and including suggested timeframes for sections within each session (content). The suggested timeframes for session activities included in the leader guide were based on the time required during simulation of selected session activities with patient partners. 

### Stakeholder partner input—survey

All five stakeholder partners completed the online survey. Stakeholder partners’ assessments of acceptability of the intervention were high, with mean ratings ranging from 4.0 to 5.0 (see Table 2) and a single open-ended comment of “I believe this manual is an excellent tool to improve quality of life for individuals with pulmonary disease. The goals and self-policing offer structure for someone developing an exercise and physical activity guide.”

**Table 2:** Stakeholder partner ratings of the physical activity counseling intervention

**Question**	**Mean**	**Range**
1. How important was the content included in this intervention for increasing physical activity in people with chronic respiratory disease? (1 = Not important, 5 = Essential)	5.0	5
2. How understandable did you find the workbook materials in this intervention? (1 = Not at all understandable, 5 = Extremely understandable)	4.8	4–5
3. How engaging did you find the learning activities and planned discussions in this intervention? (1 = Not at all engaging, 5 = Extremely engaging)	4.4	4–5
4. How well do you think that this intervention will motivate people with chronic respiratory disease to increase their physical activity? (1 = Not at all motivating, 5 = Extremely motivating)	4.6	4–5
5. How feasible will it be for people with chronic respiratory disease to carry out the required activities for this intervention? (1 = Not at all feasible, 5 = Extremely feasible)	4.0	3–5
6. How effective do you predict this intervention will be for people with chronic respiratory disease who want to increase their physical activity? (1 = Not at all effective, 5 = Extremely effective)	4.4	3–5
7. How likely would you be to recommend this intervention to a friend or family member with chronic respiratory disease? (1 = Not at all likely, 5 = Extremely likely)	5.0	5

Table 3 lists the stakeholder partners’ ratings of intervention outcome constructs. A single open-ended comment stated, “I think measuring shortness of breath and endurance should be decoupled.”

**Table 3:** Stakeholder partner ratings of outcome importance

Outcome measure	Mean	Range
1. Self-perception of physical activity, for instance, with a questionnaire	4.6	4–5
2. Measured physical activity, for instance, measuring number of steps/day	4.6	4–5
3. Belief in ability to increase physical activity or exercise (self-efficacy)	4.6	4–5
4. Motivation to exercise	4.8	4–5 1 unsure
5. Process outcomes (e.g., how often participants are setting goals for physical activity and exercise)	4.8	4–5
6. Quality of life	4.4	2–5
7. Functional exercise capacity, for instance, measuring how far and how fast someone can walk	4.2	3–5
8. Participants’ satisfaction with the intervention	4.8	4–5
9. Shortness of breath with daily tasks	4.6	4–5

In Table 3, each outcome construct was rated on a five-point Likert scale (1 = Not Important, 5 = Essential) or “unsure.” 

## Discussion

In this paper, we describe the systematic development of a PA counseling intervention for people with chronic respiratory disease that is designed to be delivered in conjunction with traditional pulmonary rehabilitation, is grounded in the HAPA framework, and successfully incorporates stakeholder input. The intervention’s strong theoretical underpinning, person-centeredness, and the contributions from the varied perspectives of stakeholders during intervention development elevate the likelihood of its feasibility, acceptability, and effectiveness in future studies. 

Theoretical underpinning is an important aspect of behavior change intervention development [25]. In other populations, theory-based health behavior change interventions have been found to be more effective than interventions not based in theory [26, 27]. In addition, having a clear foundation in health behavior change theory allows for targeted behavior change technique choices and can help guide research seeking to better understand the mechanisms behind the effectiveness of the intervention itself [28–30]. The COM-B system is an overarching framework of the essential components for behavior change (capability, opportunity, and motivation) that provide the core elements for behavior change [31]. The intervention developed in this study engages targets related to each of the three COM-B determinants of behavior change, with the HAPA framework specifically informing the motivational determinants. Using the HAPA framework as the foundation for this intervention is consistent with PA behavior change research in other populations [32–37], and yet, it is novel in the chronic respiratory disease research. Previous PA counseling interventions for people with chronic respiratory disease have not accounted for the different needs of people in the non-intender and intender categories of the HAPA model. This gap may at least partially explain the limited impact of previous interventions, and the consideration for intention level included in this intervention represents a novel improvement. 

Another strength of this intervention is its person-centeredness, which was enhanced through rich stakeholder engagement in the design phase. Health behavior changes may be more likely to occur when they match the person’s personal goals and when the person is collaboratively engaged in determining the plan for change [22]. Previously published interventions do not fully address participants’ preferences related to activity type, focus of goals, and adherence strategies [14]. These limitations limit the participants’ ability to adapt the intervention, which may contribute to their limited long-term effectiveness. In contrast, the intervention in this study provides opportunity for participants to customize their PA plans to their own situation, including their abilities (capabilities in the COM-B system), their resources (opportunities in the COM-B system), and their preferences, needs, and intentions (motivation in the COM-B system). The curriculum engages participants in setting individualized goals and plan for increasing PA to close the intention-behavior gap. In addition, this intervention allows participants to choose activities, goals, and plans that are most meaningful and feasible for them, which contrasts with other interventions that prescribe a single method of increasing PA (e.g., through walking activity) and goal setting (e.g., increasing daily step counts by a pre-set algorithm) [14]. 

Partnering with stakeholders to include their perspectives throughout the research process, including intervention development, is an important strategy to increase the acceptability, relevance, and implementation of rehabilitation interventions in various populations [38–40]. In this study, the inclusion of varied stakeholder perspectives was key for fully addressing the needs and preferences of people with chronic respiratory disease and clinicians who implement such interventions in research and clinic settings. This person-based approach may increase future value and uptake of the intervention [39, 41]. Our systematic stakeholder review of this intervention facilitated the integration of multiple points of view. For instance, the patient partners had experience with having chronic respiratory disease and intending to increase their physical activity level, consistent with the target population for this intervention. This perspective allowed them to contribute unique insights about intervention elements that would be particularly motivating to facilitate participants’ self-reflection and follow-through. These contributions likely helped shape the intervention design to meet the needs of the target participants. The clinician partner offered suggestions to increase feasibility of the intervention in a clinical environment. Last, the researcher partner brought unique expertise about existing research and suggested evidence-based changes for increasing participant motivation and maximizing fidelity to the intervention through the leader guide. We recommend that researchers and clinicians include diverse stakeholder engagement in future intervention development. In this project, we increased the feasibility of such collaboration by using videoconferencing technology, which allowed us to collaborate effectively and efficiently to mitigate barriers of varied geographic locations, scheduling limitations, and infection control concerns in the pandemic environment. 

### Limitations

While we limited the number of stakeholder partners to keep the intervention development process manageable, the small number of stakeholders included in this process may limit generalizability. The stakeholder partners’ roles as co-developers may have led to a bias toward a positive perception of the intervention. Workbook materials were designed at a seventh grade reading level, but we were unable to reduce the reading level further without eliminating or modifying core content. While the participant workbook is below the eighth to ninth grade reading level of the average adult in the USA, it is still above the recommendation of sixth grade reading level or below for patient materials [42]. 

### Implications for future research

Phase 2 testing in the ORBIT framework is a future step in investigating this intervention, including Proof-of-Concept testing (phase 2a) and progression to pilot testing (phase 2b). The leader guide and workbook materials included in this intervention provide standardized resources that will facilitate initial training and ongoing standardization of the intervention, consistent with established recommendations to enhance treatment fidelity [43]. 

While this intervention improves upon limitations in previously reported PA counseling interventions for people with chronic respiratory disease, gaps remain. First, this intervention does not target people who do not intend to change their physical activity behavior. According to the HAPA model, people in the non-intender category may benefit from interventions that primarily target change in intention. Interventions that target health behaviors of people who do not intend to change are an important area for future research using a similar approach described in this study and patient stakeholders who are “non-intenders.” Second, this intervention does not provide long-term support, which might further facilitate long-term PA behavior change. However, access to long-term support for pulmonary rehabilitation interventions such as supervised exercise and physical activity behavior change interventions is currently limited [44]. The limited duration of this intervention is consistent with current clinical practice, and therefore more feasible for clinical implementation than a longer-duration intervention. Additional research investigating the impact of interventions that are longer in duration could help inform and support advocacy for such resources. 

## Conclusion

In summary, this paper provides a detailed description of the development of a PA counseling intervention that was systematically designed using HAPA-driven behavior change techniques and stakeholder input. The intervention targets people with chronic respiratory disease who are participating in outpatient pulmonary rehabilitation and intend to increase their future PA levels. The resulting intervention provides a standardized protocol for collaborating with participants to increase their PA levels according to an individualized plan. Future research prior to randomized controlled trial of this intervention should include Proof-of-Concept testing and progression to pilot testing that will include additional investigation of intervention acceptability.

### Supplementary Information

**Additional file 1.** Screening tool to determine intention level and exercise history.

**Additional file 2.** Workbook to be issued to participants in the intervention for use during PA counseling intervention sessions.

**Additional file 3.** Leader guide to provide clinician group leader with guiding principles and instructions for leading this PA counseling intervention.

## Data Availability

The datasets used and/or analyzed during the current study are available from the corresponding author on reasonable request.

## Abbreviations

COPD: Chronic obstructive pulmonary disease
COVID-19: Coronavirus disease of 2019
CRD: Chronic respiratory disease
HAPA: Health Action Process Approach
ORBIT: Obesity-Related Behavioral Intervention Trials
PA: Physical activity
REDCap: Research Electronic Data Capture
