# Supplementary material for: Development of a physical activity counseling intervention for people with chronic respiratory disease based on the health action process approach
Source: Pilot Feasibility Stud. 2023 Oct 12;9:173. doi: 10.1186/s40814-023-01397-w (PMC10568913; doi:10.1186/s40814-023-01397-w)
Supplement: Supplementary file 1 — Additional file 1. Screening tool to determine intention level and exercise history. [file 40814_2023_1397_MOESM1_ESM.docx]

Physical Activity Planning Group – Screening for Eligibility

- Participants in this intervention group should have completed at least some of Phase II Pulmonary Rehabilitation (PR), including the Education session focused on exercise.
- Phase II PR Clinicians will screen potential participants for eligibility in this group as early as possible and no later than halfway through Phase II PR and make referral to the group as appropriate.
- In order to understand each potential participant’s recent exercise history as well as current motivation to increase physical activity and exercise levels, potential participants should complete the following questions.
- This intervention is targeted at people who are in the “Intender” phase of the Health Action Process Approach. Therefore, people who answer a or b to question 4 are eligible to participate in this group.

Screening Questions:

First, we’d like to get an idea of how much you exercise:

1. Which of the following statements best describes you?
2. I have been exercising regularly (at least 1x/week) outside of Phase II Pulmonary Rehab sessions for more than 6 months.
3. I have been exercising regularly (at least 1x/week) outside of Phase II Pulmonary Rehab sessions for less than 6 months.
4. I am not currently exercising outside of Phase II Pulmonary Rehab sessions.

If you answered a or b to #1, please answer questions 2-3:

1. How often do you perform exercise that feels at least “somewhat hard”

(at least 12-14/20) on the scale below?

______ sessions/week

| **Borg Rating of Perceived Exertion Scale** | |
| --- | --- |
| **Score** | **Level of Exertion** |
| 6 |  |
| 7 | Very, Very Light |
| 8 |  |
| 9 | Very Light |
| 10 |  |
| 11 | Fairly Light |
| 12 |  |
| 13 | Somewhat Hard |
| 14 |  |
| 15 | Hard |
| 16 |  |
| 17 | Very Hard |
| 18 |  |
| 19 | Very, Very Hard |
| 20 |  |

1. How long do you exercise in a typical exercise session?

______ minutes

Now we’d like to learn about what your plans are for exercise.

1. Which of the following statements best describes your current plans for exercise?
   1. I intend to increase the amount or intensity of physical activity and exercise that I do in the next 30 days.
   2. I intend to increase the amount or intensity of physical activity and exercise that I do in the next 6 months.
   3. I intend to keep my level of physical activity and exercise the same in the next 6 months.
   4. I intend to decrease my level of physical activity and exercise in the next 6 months

Interpretation:

ATS/ERS exercise recommendations for people in pulmonary rehabilitation (similar to AACVPR guidelines):

F: 3-5x/week

I: Vigorous exercise (>60% max work rate or 12-14/20 RPE)

T: 20-60 min

Garvey C, Bayles MP, Hamm LF, et al. Pulmonary rehabilitation exercise prescription in chronic obstructive pulmonary disease: review of selected guidelines. An official statement from the American Association of Cardiovascular and Pulmonary Rehabilitation. *J Cardiopulm Rehabil Prev.* 2016;36:75-83.

Participants can be categorized as follows:

|  | Answered a or b to #4? Y | Answered a or b to #4? N |
| --- | --- | --- |
| No exercise | *Intender* | *Non-Intender* |
| Some exercise but less than PR guidelines based on answers to #2 and #3 (<3sessions/week OR <20min/session at >12/20 RPE) | *Actor (intending to increase)* | *Actor (no intention to increase)* |
| Meets or exceeds PR guidelines based on answers to #2 and #3 (<3sessions/week OR <20min/session at >12/20 RPE) | *Actor (intending to increase)* | *Actor (no intention to increase)* |

Green = eligible for this Physical Activity Intervention

Red = ineligible for this Physical Activity Intervention
