# Supplementary material for: Development of a physical activity counseling intervention for people with chronic respiratory disease based on the health action process approach
Source: Pilot Feasibility Stud. 2023 Oct 12;9:173. doi: 10.1186/s40814-023-01397-w (PMC10568913; doi:10.1186/s40814-023-01397-w)
Supplement: Supplementary file 2 — Additional file 2. Workbook to be issued to participants in the intervention for use during PA counseling intervention sessions. [file 40814_2023_1397_MOESM2_ESM.docx]

Physical Activity Planning Group

Workbook

Welcome to the Physical Activity Planning Group! Now that you’ve started Pulmonary Rehabilitation, you’re ready to start planning what life-long physical activity and exercise will look like for you. In this five-session class, you’ll explore ways to add physical activity and exercise into your routine long after Pulmonary Rehabilitation is over.

Before your first class, please complete the homework on page 2.

Physical Activity Planning Group

Session 1 Homework

Physical Activity and Exercise:

What do these terms mean to you?

Physical Activity: _____________________________________________________

Exercise: ___________________________________________________________

Exercise Effects:

Do you exercise now or in the past? If so, how has it helped you?

If you start exercising more now, how do you think that will impact your health and well-being?

What concerns do you have about adding more exercise into your life?

How important do you think exercise is for you on a scale of 0-10?

0 1 2 3 4 5 6 7 8 9 10

Not Important Extremely Important

Physical Activity Planning Group

Workbook

Session 1:

Agenda:

- Introductions
- Group Guidelines
- Physical Activity and Exercise: Why should I and how can I do it?
  - Homework Review
- Exploring Change

Purpose:

The purpose of this group is to support you as you explore how you will make changes in your level of physical activity. Over the next five sessions we’ll look at what exercise and physical activity are and how they can impact your life. You’ll set goals for yourself, and you’ll have the chance to make and carry out your own plan around exercise and physical activity.

Group Guidelines:

In this group, we’ll work together to make positive changes in your physical health. Some guidelines for how our group can best work together are:

- Our focus will be on looking forward and making things better rather than looking back at what has been wrong.
- It will be up to each person to decide what and how they will change.
- What each person shares with the group will not be shared outside of the group.

What other guidelines would you like to set?

Changing Exercise and Physical Activity

Change is hard! How has exercise gone for you in the past? Are there times where you were able to increase your physical activity? If so, what factors helped you succeed?

Group Activity: Four Square

In this activity, your group will make lists of the pros and cons of increasing your physical activity level with exercise. You will also make lists of the pros and cons to not increasing your physical activity level.

|  | Pros | Cons |
| --- | --- | --- |
| Increase physical activity and exercise |  |  |
| Keep doing what you’re doing |  |  |

As you review these lists of pros and cons, what is one item that feels meaningful to you?

How can you adjust your exercise plan with that item in mind?

Now, think of a future where you are adding exercise and physical activity into your routine.

Discuss with the group:

How will your life be different? For instance, what’s one thing you might enjoy doing if you were more active than you are now?

What will it take to get there?

Physical Activity Planning Group Workbook

Session 2 Homework

In Session 2, you’ll make a plan to start doing more physical activity. To prepare, brainstorm ways that you could be more physically active. You don’t need to commit to any of these ideas right now! Your goal now is just to make a list of ideas.

What are three ways that you could exercise?

1.

2.

3.

What are three ways that you could be more physically active in your day-to-day life?

1.

2.

3.

Physical Activity Planning Group

Workbook

Session 2

Agenda:

- Physical Activity and Exercise: Information
- Physical Activity Planning

Physical Activity and Exercise: Information

It’s time to start making a plan for your physical activity. You have likely already received information from your healthcare team. It may also be helpful to know what guidelines exist related to physical activity and exercise. But remember: what steps you take are up to you!

**Physical Activity Guidelines:**

From the Physical Activity Guidelines for Americans: “Adults should move more and sit less throughout the day. Some physical activity is better than none.”

<https://health.gov/our-work/physical-activity/current-guidelines>

The most common and effective way to increase physical activity is to keep track of how many steps you take each day with a pedometer or smartphone and aim to increase that number.

You can also increase your physical activity in other ways:

- Park your car further away from your destination in order to increase the distance you will walk.
- Take the stairs rather than an elevator.
- Walk or bike somewhere rather than drive.
- Leave your home at least one time every day.

**Cardiovascular Endurance Exercise:**

**General Recommendation:** Cycling or walking 3-5x/week for 20-60 minutes per session at high intensity. Exercise may be completed in shorter intervals, if needed.

High intensity is defined as: 60-80% of peak work rate, 4-6/10 on modified Borg Scale for Dyspnea or 12-14/20 on Borg Rating of Perceived Exertion Scale.

So, what does this mean? Let’s break it down:

| **Peak work rate**: | **4-6/10 on the modified Borg Scale for Dyspnea:** | **12-14/20 on the Borg Rating of Perceived Exertion Scale:** |
| --- | --- | --- |
| This refers to the hardest work that you can do, even for just 30 seconds. Imagine what this is like. Now think about exercising at about  1/2 – 3/4 of that intensity. | This means that you exercise hard enough to experience somewhat severe to severe shortness of breath during exercise. | This means that you exercise with a fairly light to somewhat hard amount of effort. |

Please use whichever definition above makes the most sense for you to guide your endurance exercise as we progress through this program!

You can use one of the two scales on the next page to rate how hard you are working (Rating of Perceived Exertion – or RPE – scale on the left) and how short of breath you are (Dyspnea Scale on the right) during exercise.

| **Borg Rating of Perceived Exertion Scale** | |
| --- | --- |
| **Score** | **Level of Exertion** |
| 6 |  |
| 7 | Very, Very Light |
| 8 |  |
| 9 | Very Light |
| 10 |  |
| 11 | Fairly Light |
| 12 |  |
| 13 | Somewhat Hard |
| 14 |  |
| 15 | Hard |
| 16 |  |
| 17 | Very Hard |
| 18 |  |
| 19 | Very, Very Hard |
| 20 |  |

*Borg GA.* Psychophysical bases of perceived exertion.*Med Sci Sports Exerc.*1982;14:377–81.

| **Modified Borg Dyspnea Scale (for shortness of breath)** | |
| --- | --- |
| **Score** | **Shortness of Breath** |
| 0 | Nothing at all |
| 0.5 | Very, very slight (just noticeable) |
| 1 | Very slight |
| 2 | Slight (light) |
| 3 | Moderate |
| 4 | Somewhat Severe |
| 5 | Severe (heavy) |
| 6 |  |
| 7 | Very severe |
| 8 |  |
| 9 |  |
| 10 | Very, very severe (almost max) |
| * | Maximal |

Mahler DA, Horowitz MB. Perception of breathlessness during exercise in patients with respiratory disease. *Med Sci Sports Exerc.* 1994;26:1078-1081.

**Resistance Training:**

Another type of exercise you may want to use in your daily routine is resistance training. Resistance training is any type of exercise where you are pushing, pulling, or lifting against a weight or object. Examples are:

- lifting weights such as dumbbells
- using exercise machines such as a chest press or leg press
- moving your arms or legs against an elastic band
- moving your body weight, for instance with a push-up or sit-up

**General recommendation:** Do resistance training exercises for 8-12 repetitions of each exercise 2-3x/week.

When deciding on the details of physical activity and exercise, it can be helpful to use the “FITT principle” to describe the following details of how you will exercise:

Frequency: How often?

Intensity: How hard will you work? You can describe the level of effort or workload or both.

Time: How long will you engage in the activity?

Type: What physical activity/exercise will you do? Be specific!

| **Cardiovascular Exercise Example** | **Physical Activity Example:** |
| --- | --- |
| Frequency: 4x/week  Intensity: RPE of 13/20  Time: 30 minutes  Type: Walking outside | Frequency: every hour  Intensity: low intensity (8/20)  Time: 2 minutes  Type: Stand up and walk in my home |

Physical Activity Planning

What is one thing you will do to increase your exercise or physical activity in the next week?

**What will you do?** (consider frequency, intensity, time, and type of activity)

**Where will you do this?**

**When will you do this?**

How confident are you that you can complete your physical activity plan above?

0 1 2 3 4 5 6 7 8 9 10

Not Confident Extremely Confident

If you chose anything less than a 10, what can you do today to increase this number?

Homework: Complete your physical activity plan for the next week!

Physical Activity Planning Group Workbook

Session 3 Homework:

Your first homework is to complete your physical activity plan from Session 2! Also, before Session 3, please take a few minutes to think about your goals by reading page 14 and completing the worksheet on page 15.

Setting Goals

Setting SMART goals is a method to describe what you want to achieve. When making a change, having a target can help you:

- stay motivated
- decide on an action plan
- know when you have achieved your goal

**A SMART goal is:**

**S**pecific: What actions will you take?

**M**easurable: How will you measure whether you have met the goal?

**A**chievable: Is the goal doable?

**R**elevant: Is the result important to you?

**T**ime-bound: When do you plan to meet the goal?

*Examples*

*#1: I will walk for 30 minutes, making it at least 2 laps around the park (1/2 mile) in that time, 4 out of 7 days this week.*

*#2: I will ride a stationary bike at 10mph on level 1 resistance for 20 minutes, at least 3 out of 7 days/week, for the next month.*

*#3: I will take at least 7,000 steps per day every day for the next week.*

*#4: Every time I leave and enter my apartment without carrying anything, I will take the stairs instead of the elevator for the next month.*

**Goal Setting Activity:**

Set at least two SMART goals that describe the physical activity and/or exercise that you will do. One goal should describe what you aim to do in the next week and one goal should describe what you aim to do in the next 2 weeks.

**Goal #1:** In the next week, I will:

Name each of the FITT principles included in your goal:

Frequency:

Intensity:

Time:

Type:

**Goal #2:** In the next 2 weeks, I will:

Name each of the FITT principles included in your goal:

Frequency:

Intensity:

Time:

Type:

Physical Activity Planning Group

Workbook

Session 3:

Agenda:

- Goal Setting with SMART Goals
  - Homework Review
  - Thinking about “Why?”
- Making a Plan
- Identify Resources
- Self-efficacy

Goal Setting – Thinking about “Why?”

After we review the goals that you set in the homework together, let’s take a few minutes to reflect on why we’re working on increasing physical activity and exercise in the first place.

**How will your life be better if you increase your physical activity level as you describe in your goals?** Name at least one thing you might enjoy doing if you were more physically active than you are now:

Why are those things important to you?

Making a Plan

What is your plan for increasing your physical activity or exercise in the next two weeks?

**What will you do?** (refer to your goals on p. 15!)

*Example: I will walk for 30 minutes, making it at least 2 laps around the park (1/2 mile) in that time, 4 out of 7 days this week.*

**Where will you do this?**

*Example: In the park near my house*

**When will you do this?**

*Example: On Monday, Wednesday, Friday, and Saturday mornings*

Resources

Now let’s list the resources that you have to help you be more active. Resources can be physical, interpersonal, or intrapersonal.

Physical Resources:

Physical Resources are things you have around you that can help you increase your physical activity level. Examples of physical resources include:

- exercise equipment
- a walking path near your home
- a community center that offers exercise classes that you enjoy
- music to listen to while you are active
- a smart phone that will help you track exercise or steps per day
- a diary or calendar to keep track of your steps or exercise

Interpersonal Resources:

Interpersonal resources are other people who can help you increase your physical activity level. Examples of interpersonal resources include:

- exercise partner(s)
- supportive family members or friends
- members of this group

Intrapersonal Resources:

Intrapersonal resources are characteristics within you that can help you increase your physical activity level. Examples include:

- experience with physical activity (for example, past success with exercise or sports)
- knowledge about physical activity (for example, your training in Phase II Pulmonary Rehab will help you know what to do)
- persistence
- courage
- creativity

What resources do you have that will help you increase your physical activity and exercise?

Self-Efficacy

Self-efficacy is your belief in your ability to complete a task. Let’s explore your self-efficacy for increasing your physical activity and exercise.

You have already completed exercise in your Pulmonary Rehab sessions. How confident are you that you can continue to exercise on your own outside of Pulmonary Rehab in the next 2 weeks?

0 1 2 3 4 5 6 7 8 9 10

Not confident Extremely confident

If you chose anything less than a 10, what would it take to increase this number?

Now that we’ve completed Session 3, your next step is to complete the physical activity plan that you came up with in today in order to achieve your goals!

Physical Activity Planning Group Workbook

Session 4 Homework:

Your main homework is to complete your physical activity plan!

In the day before Session 4, also complete the questions on pages 22-23 below:

What physical activity did you actually do in the last two weeks? How does what you actually did compare to your plan on p. 18?

If you were successful in enacting your plan from Session 3, Congratulations!

If you didn’t achieve some of your plan, why not? And how could you adapt your plans in the future?

Goal Setting

Now it’s time to decide whether the goals and plan that you set in Session 3 are on target! If you weren’t able to achieve your physical activity plan since the last session, are the goals you set attainable? This is the time to raise or lower your goals as you see fit. Write new goals for the next month:

**Goal #1:** In the next month, I will:

Name each of the FITT principles included in your goal:

Frequency:

Intensity:

Time:

Type:

___________________________________________________________________

**Goal #2:** In the next month, I will:

Name each of the FITT principles included in your goal:

Frequency:

Intensity:

Time:

Type:

___________________________________________________________________

Finally: Why are these goals important to you? How will your life be better if you meet these goals?

Physical Activity Planning Group

Workbook

Session 4:

Agenda:

- Action Planning
  - Discuss Progress (p. 22 homework)
  - Identify Challenges
  - Update Goals (p. 23 homework) and Action Plan
- Self-Efficacy
- Coping Planning

Physical Activity Planning - Updates

Welcome back! Two weeks ago, you came up with a plan to increase your physical activity. We’ll start this session with a discussion about how it went by reviewing the homework on p. 22.

Challenges

What challenges did you face since the last session that made physical activity more difficult or prevented you from doing it altogether?

What are some ways that you could conquer these challenges?

What resources were helpful to you as you increased your physical activity last week? *Remember that your resources can be physical (e.g., equipment, space), interpersonal (e.g., supportive friends or family), or intrapersonal (personal traits that help you succeed).*

Update Goals and Action Plan

For homework, you updated your goals and extended them to one month. After we talk about the groups’ updated goals, we’ll make a plan to help you achieve your goals.

What is your plan for increasing your physical activity or exercise in the next month?

**What will you do?**

Frequency:

Intensity:

Time:

Type:

**Where will you do this?**

**When will you do this?**

Self-Efficacy

It is important to maintain your increases in physical activity and exercise long-term in order to see progress. In the following two pages, we’ll explore ways that you can boost your belief in your ability to keep your plan going.

How confident are you that you can keep exercising for the next month?

0 1 2 3 4 5 6 7 8 9 10

Not Confident Extremely Confident

If you chose anything less than a 10, why is that and is there anything you can do today to increase this number?

How confident are you that you can continue with this plan in the next month:

Even if you are busy, tired, or not in the mood?

0 1 2 3 4 5 6 7 8 9 10

Not Confident Extremely Confident

Even when you cannot see positive changes right away?

0 1 2 3 4 5 6 7 8 9 10

Not Confident Extremely Confident

Even when you face difficulties or unexpected events?

0 1 2 3 4 5 6 7 8 9 10

Not Confident Extremely Confident

If you chose anything less than a 10 for these three questions, is there anything you can do today to increase these numbers?

Coping Planning

You now have a solid plan for increasing your physical activity. *Excellent!*

Even with a good plan, setbacks may occur. Planning for these setbacks will ensure your success. First, we’ll explore what problems are most likely to arise.

Activity:

In the left column, name up to three challenges to physical activity that you faced that are most likely to hinder your physical activity plan in the future. In the right column, describe how you can cope with each barrier or challenge and get back on track for physical activity.

| **Barrier or Challenge** | **Coping Strategy** |
| --- | --- |
| 1. |  |
| 2. |  |
| 3. |  |

Plan Recovery:

Imagine that you have not been exercising for a while.

How confident are you that you will be able to resume your physical activity if you miss one session of exercise or physical activity?

0 1 2 3 4 5 6 7 8 9 10

Not Confident Extremely Confident

How confident are you that you will be able to resume your physical activity if you miss some sessions of exercise or physical activity?

0 1 2 3 4 5 6 7 8 9 10

Not Confident Extremely Confident

Which coping strategies from the last page will be most helpful as you resume your physical activity plan?

Congratulations! You now have a complete Physical Activity Plan in place with 1) goals, 2) a specific action plan, and 3) a plan for how to recover if you stop your action plan. We’ll meet again in 4 weeks to make any final changes to your action and recovery plans. We’ll also talk about how to progress your physical activity plan. In the meanwhile, enjoy your more active lifestyle!

Physical Activity Planning Group Workbook

Session 5 Homework:

Before Session 5, please take a few minutes to think about how your plan from the last session has gone by answering the questions below.

**Physical Activity Plan:** First, let’s check on where you are at on your plan:

What was your plan from the last session (see p. 26)? (re-write here)

**What will you do?**

Frequency:

Intensity:

Time:

Type:

**Where will you do this?**

**When will you do this?**

How closely did you follow your action plan since the last session?

0 1 2 3 4 5 6 7 8 9 10

I followed 0% of my plan I followed 100% of my plan

What (if any) new challenges did you find? What challenges do you anticipate you will find in the future?

What resources were most helpful as you completed your physical activity action plan? *(Refer back to Session 3, p. 20, for a reminder of the resources that you listed.)*

Did you have any lapses in completing your Physical Activity Action Plan? If so, what Coping Strategies were most helpful?

Physical Activity Planning Group

Workbook

Session 5:

Agenda:

- Updates:
  - Action Plan (homework review)
  - Coping Plan (homework review)
  - Resources and Challenges (homework review)
- Exercise Progression
- Advanced Goal Setting
- Wrap-Up

Exercise Progression

As your physical abilities improve, it’s important to progress your physical activity in order to obtain the most benefit. Here are some examples of ways to increase your physical activity:

Total physical activity in a day:

- Increase total number of steps per day

Cardiovascular exercise:

- Increase intensity of exercise (faster speed, more resistance, add incline)
- Increase duration of exercise (more minutes of exercise per session)
- Increase frequency of exercise (increase # of exercise sessions per week)

Strengthening exercise:

- Increase resistance (heavier weight, more difficulty body position)
- Increase number of repetitions

These changes should be added slowly to avoid injury and build on past success. As you get stronger and more fit:

a) How will you know when it is time to progress your physical activity?

b) How will you progress your exercise and what parts can you increase?

Advanced Goal Setting

Once you have achieved each goal, it is time to set a new goal. Goals should continue to be in a SMART goal format.

How will you continue to remember what your SMART goals are?

How often will you check the status of your goals and write new goals, and how will you remember to do so?

Let’s practice progressing one of your goals.

Re-write one of your goals here:

When you achieve this goal, what is one way that you could write a new goal that builds on this success?

Congratulations! You have created a Physical Activity Action Plan that sets you up for long-term success with increasing your physical activity levels. We wish much success with plenty of improvement in your physical ability, symptoms, physical and mental health, quality of life, and overall confidence to show for it!
