# Supplementary material for: Development of a physical activity counseling intervention for people with chronic respiratory disease based on the health action process approach
Source: Pilot Feasibility Stud. 2023 Oct 12;9:173. doi: 10.1186/s40814-023-01397-w (PMC10568913; doi:10.1186/s40814-023-01397-w)
Supplement: Supplementary file 3 — Additional file 3. Leader guide to provide clinician group leader with guiding principles and instructions for leading this PA counseling intervention. [file 40814_2023_1397_MOESM3_ESM.pdf]

# Physical Activity Planning Group
# Group Leader Instructions

## Table of Contents:


1. Overview
   a. Class Format
   b. Guiding Principles

2. Session 1

3. Session 2

4. Session 3

5. Session 4

6. Session 5

7. Appendix A: Example Completed Four Square Activity

8. Appendix B: Exercise and Physical Activity Logs

9. Appendix C: Example Community-Specific Resources


From: Tappan RS, Ettinger JR, Mahon D, et al. Development of a physical activity counseling intervention for people with chronic respiratory disease based on the Health Action Process Approach. Pilot and Feasibility Studies. 2023; in press. https://doi.org/10.1186/s40814-023-01397-w

## Overview

It is common for people with chronic respiratory disease to make meaningful improvements in function, symptoms, and quality of life when they participate in exercise training in pulmonary rehabilitation. Unfortunately, after pulmonary rehabilitation is over, they often stop or decrease their physical activity and exercise and ultimately lose the valuable gains that they have made.

The aim of this class series is to collaborate with people who are enrolled in pulmonary rehabilitation to develop and implement a plan for increasing their participation in physical activity and exercise long after pulmonary rehabilitation has ended.

## Class Format

Participant Eligibility:
Participants in Phase II Pulmonary Rehabilitation should be screened for intention to increase their physical activity level prior to enrolling in this class series using the document "Physical Activity Planning Screening Tool." Ensuring that participants have an intention to increase their physical activity and/or exercise at baseline will allow for a productive experience.

Class Structure:
This class is delivered in five one-hour sessions and a group format of up to 6 people in each group. Some common elements to include in each class are:

- Each class session has homework that each participant is meant to complete 1-2 days prior to the class session. Remind participants at the end of each session to complete the homework for the next session. Consider providing an additional homework reminder 1-2 days before each session.
- Each session should start with a brief summary of the previous session and objectives for the current session.
- Each session should end with a summary of what was discussed, including specific themes highlighted in individuals' responses.
- The Patient Workbook that accompanies this class should be used as a guide for the content and activities in each session, and the participants should be oriented to the Workbook throughout the class sessions. Flexibility in the content, areas of focus, and timing may be necessary based on participant responses and ability to read and understand the content independently.

Timing:

- Participants should start this class series when they are midway through Phase II Pulmonary Rehabilitation with Sessions 4 and 5 occurring after Phase II Pulmonary Rehabilitation has ended, if possible. This timing will allow them to have had sufficient education about what exercise is and training in how to exercise safely and effectively. It will also allow them the opportunity to problem-solve any barriers to their plan that become apparent after their structured Phase II Pulmonary Rehabilitation program has ended.
- Sessions 1-3 should be delivered one time per week, Session 4 should be 2 weeks after Session 3, and Session 5 should be 4 weeks after Session 4.

Group Leader Training:
Group Leaders should have training in the principles and application of exercise and physical activity as well as chronic pulmonary disease as well as experience leading group discussions and activities, consistent with providers and trainees working in Pulmonary Rehabilitation. Prior to leading these sessions, new Group Leaders should undergo live/synchronous training that includes practice of leading simulated group interactions with feedback. The following written materials are provided as an additional resource.

## Guiding Principles

1. This class will focus on collaborating with people as they develop their plan for increased physical activity rather than teaching them about what they are "supposed" be doing.
2. This class will focus on each person making their own plan for the future rather than dwelling on what other people think they should do or what has been wrong in the past.
3. In the spirit of collaboration, the following skills should be used:
    a. Open-ended, probing questions: Guiding open-ended questions are included in the Patient Workbook that accompanies this class. Follow-up questions will be appropriate at times as well.
    b. Active listening skills: Active listening will help build group trust and rapport and improve group leader and participant understanding. Active listening should be used frequently and modeled by the group leader. Specific active listening skills that may be useful include:
        i. Restating: paraphrase what the other person said to demonstrate that you are listening
        ii. Summarizing: summarize multiple facts/ideas to check understanding
        iii. Reflecting: state your impression of how the other person is feeling
        iv. Silence: allow for silence to allow time to think and space to talk
    c. Motivational interviewing principles have been incorporated into the plan and instructions for each session. Additional training in Motivational Interviewing may be helpful for group leaders as well.
4. Sharing of information: While open communication is conducive to group collaboration, sharing of personal information by group participants is not required. Information that is shared should be kept confidential by both the group leader and participants.
5. This class is designed using the Health Action Process Approach (HAPA) as the foundational framework for facilitating behavior change. More information about HAPA is available here: http://www.hapa-model.de
6. Behavior change techniques are listed according to the Behaviour Change Technique Taxonomy (v1), Appendix 4 in: Michie S, Atkins L, West R. Behavior Change Wheel/Behavior Change Technique Taxonomy. Silverback Publishing; 2014.

Additional Resources for Motivational Interviewing

- Wagner CC, Ingersoll KS. Motivational Interviewing in Groups. Guilford Press; 2013.
- Miller WR, Rollnick S. Motivational Interviewing: Helping People Change. 3rd ed. Guilford Press; 2013.
- Motivationalinterviewing.org

# Session 1

Session 1 is divided into 3 sections: 1) Introductions and Establishing Group Guidelines, 2) Discussing Physical Activity and Exercise, 3) Exploring Change.

**Section I: Introductions and Establishing Group Guidelines, Workbook page 4**

(Suggested Time: 15 minutes)

1. Introduce the purpose of the group in your own words. For example:

    *Welcome! You've all been participating in Phase II Pulmonary Rehabilitation, and you have learned about physical activity and exercise through the education session about exercise and from your exercise sessions with Physical Therapy. Now it's time to start thinking about how you will manage your physical activity and exercise after Pulmonary Rehab ends.*

    *We'll meet for up to 60 minutes each session as we explore these questions together. There will be homework before each session, and our focus in these sessions will be on creating and implementing a plan for physical activity that works for each of you.*
2. The group leader and participants each introduce themselves and share their hopes and goals for the group.
3. Lead a group discussion to achieve consensus on the ground rules, based on the three guidelines given in the Workbook plus any additional guidelines that the group members feel are important. Write these guidelines down and keep them in a prominent place for each session. Examples include:
    a. maintaining confidentiality within the group
    b. being respectful of each other's experiences and values
    c. turn-taking
    d. consistent attendance
    e. participation from everyone in the discussion
    f. whether or not the group leader will make cold calls

Page 4

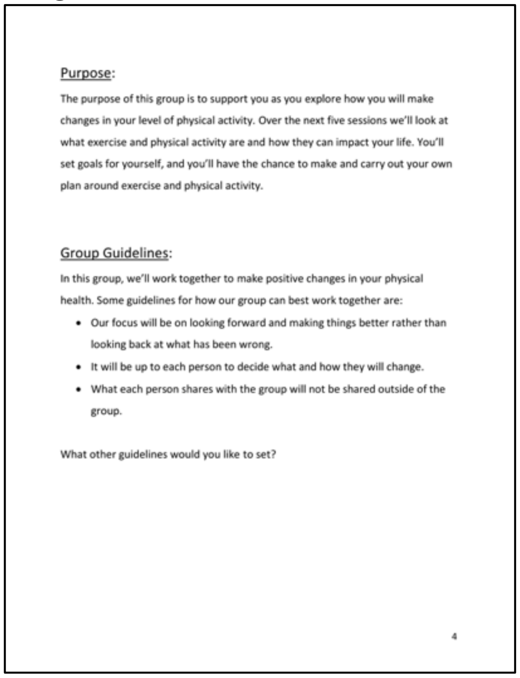

Purpose:

The purpose of this group is to support you as you explore how you will make changes in your level of physical activity. Over the next five sessions we'll look at what exercise and physical activity are and how they can impact your life. You'll set goals for yourself, and you'll have the chance to make and carry out your own plan around exercise and physical activity.

Group Guidelines:

In this group, we'll work together to make positive changes in your physical health. Some guidelines for how our group can best work together are:

- Our focus will be on looking forward and making things better rather than looking back at what has been wrong.
- It will be up to each person to decide what and how they will change.
- What each person shares with the group will not be shared outside of the group.

What other guidelines would you like to set?

4

Additional File #3

**Section II: Discussing Physical Activity and Exercise, Workbook page 2**
(Suggested Time: 15 minutes)
HAPA elements addressed: Outcome Expectancies, Risk Perception
Behavior Change Techniques included: Comparative Imagining of Future Outcomes, Focus on Past Success

The objective of this section is to explore participants' beliefs about risks and likely outcomes of increasing their physical activity level. Participants should have already answered the questions on Workbook page 2 for homework, and discussion should center their responses to these questions.

The World Health Organization definitions of "physical activity" and "exercise" are below for your reference as you discuss what these terms mean to the participants:

- Physical activity: any bodily movement produced by skeletal muscles that requires energy expenditure.
- Exercise: physical activity that is "planned, structured, repetitive, and purposeful in the sense that the improvement or maintenance of one or more components of physical fitness is the objective."

When discussing participants' ratings of how important they think exercise is for them, a useful probing follow-up question that highlights the positive aspects of exercise may be "Why is your rating a *(insert participant's stated score here)* rather than a 0?"

Page 2

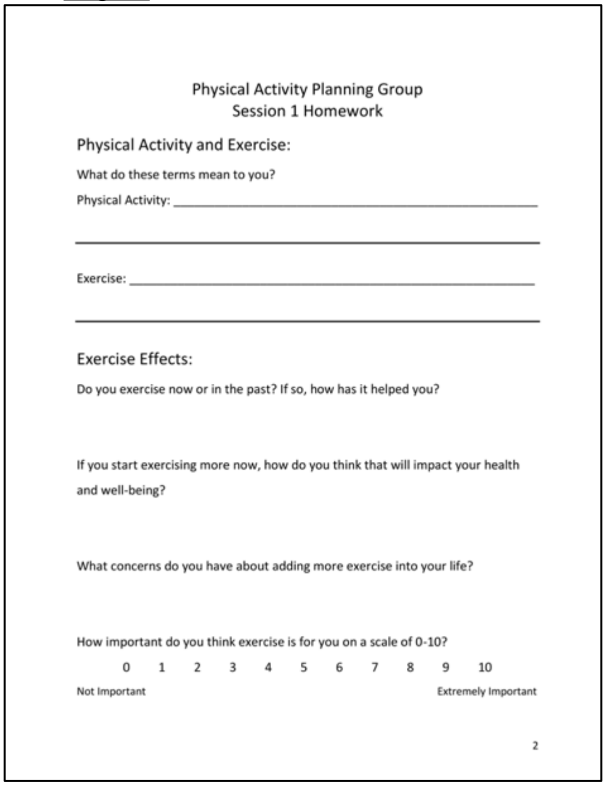
Physical Activity Planning Group
Session 1 Homework

Physical Activity and Exercise:

What do these terms mean to you?

Physical Activity: ____________________

____________________

Exercise: ____________________

____________________

Exercise Effects:

Do you exercise now or in the past? If so, how has it helped you?

If you start exercising more now, how do you think that will impact your health and well-being?

What concerns do you have about adding more exercise into your life?

How important do you think exercise is for you on a scale of 0-10?

0 1 2 3 4 5 6 7 8 9 10

Not Important Extremely Important

2

**Section III: Exploring Change, Workbook pages 5-6**
(Suggested Time: 25-30 minutes)
HAPA elements addressed: Task and Maintenance Self-Efficacy, Outcome Expectancies, Risk Perception
Behavior Change Techniques included: Pros and Cons, Comparative Imagining of Future Outcomes, Focus on Past Success

The objective of the first set of questions is to boost Task Self-Efficacy by exploring times in the past where increasing physical activity went well and what factors led to success.

The objective of the second activity (Four Square) is to explore ambivalence toward change. It is common for people to feel two ways about making a life change at the same time – wanting to make a change, and yet hesitant to make a change. In this discussion, focus on the reasons to increase physical activity level. Here are the steps:

1. Complete the four square form with participants, marking responses in a common location, such as a whiteboard. Include everyone's answers as the group brainstorms a list of responses. An example of a completed form is available in Appendix A.
2. Participants enter the items from each square that feel most impactful to them in the Four Square form their Workbook on page 5 as well.

Page 5

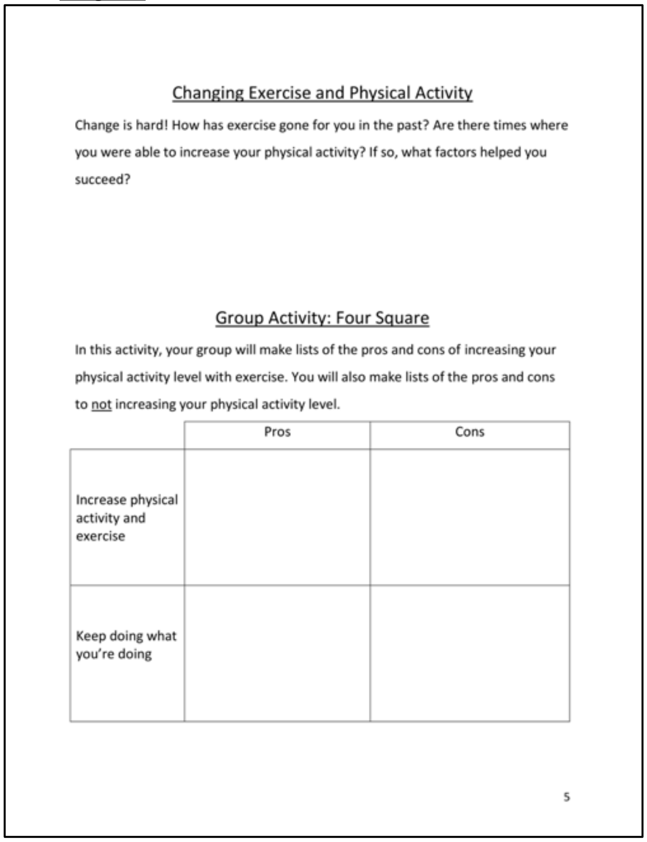

Changing Exercise and Physical Activity

Change is hard! How has exercise gone for you in the past? Are there times where you were able to increase your physical activity? If so, what factors helped you succeed?

Group Activity: Four Square

In this activity, your group will make lists of the pros and cons of increasing your physical activity level with exercise. You will also make lists of the pros and cons to not increasing your physical activity level.

| | Pros | Cons |
|---|---|---|
| Increase physical activity and exercise | | |
| Keep doing what you're doing | | |

5

3. Each person writes an item that feels meaningful to them on Workbook page 6.
4. Discuss as a group how understanding these pros and cons can help support their choice to increase physical activity and exercise.
    a. If a person chooses a pro for exercise (upper left hand corner) or a con for the status quo (lower right hand corner), discuss how they can capitalize on this information. For example, if someone says that exercising would help them walk longer distances (pro for exercise), then they may be able to add a walk with a friend or family member into their exercise routine.
    b. If a person chooses a con for exercise (upper right hand corner) or a pro for the status quo (lower left hand corner), ask how they can overcome that thing in order to support a change to increase physical activity level. For instance, if someone says that exercise takes a lot of time, then they could overcome that by incorporating exercise into something that they are already doing (e.g., walking their dog at a faster speed).
5. When discussing the 2nd question on Workbook page 6 (How can you use an item on this list to support increasing your physical activity and exercise?), highlight strategies to increase the likelihood and positive impact of increasing physical activity level.

The objective of the final set of questions is 1) to focus on expected positive outcomes of increasing physical activity and/or exercise and 2) to consider the steps that may increase participants' confidence in their ability to achieve and maintain this life change.

<u>Page 6</u>

As you review these lists of pros and cons, what is one item that feels meaningful to you?

How can you adjust your exercise plan with that item in mind?

Now, think of a future where you are adding exercise and physical activity into your routine.
Discuss with the group:
How will your life be different? For instance, what's one thing you might enjoy doing if you were more active than you are now?

What will it take to get there?

6

# Session 2

The objective of Session 2 is for participants to learn about physical activity and exercise recommendations and develop an initial plan for increasing physical activity and exercise. By the end of today's session, participants should have a plan of doing one thing to increase their physical activity level over the next week. Participants should have already started brainstorming ideas in their homework on Workbook page 7.

**Section 1: Physical Activity and Exercise Information, Workbook pages 9-12**
(Suggested Time: 25-30 minutes)
HAPA elements addressed: Action Planning
Behavior Change Techniques included: Instruction on How to Perform a Behavior

Review the information in this section with the participants, answering questions as needed. Use information from the clinician team in the Pulmonary Rehab program as a means to communicate recommended exercise prescription to the participants as needed.

Pages 9-12

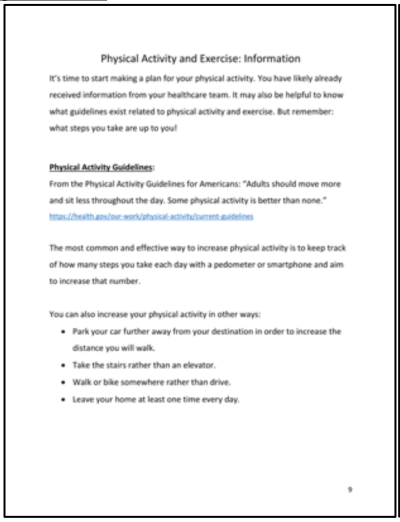

Physical Activity and Exercise: Information

It's time to start making a plan for your physical activity. You have likely already received information from your healthcare team. It may also be helpful to know what guidelines exist related to physical activity and exercise. But remember: what steps you take are up to you!

**Physical Activity Guidelines:**
From the Physical Activity Guidelines for Americans: "Adults should move more and sit less throughout the day. Some physical activity is better than none."
https://health.gov/our-work/physical-activity/current-guidelines

The most common and effective way to increase physical activity is to keep track of how many steps you take each day with a pedometer or smartphone and aim to increase that number.

You can also increase your physical activity in other ways:
- Park your car further away from your destination in order to increase the distance you will walk.
- Take the stairs rather than an elevator.
- Walk or bike somewhere rather than drive.
- Leave your home at least one time every day.

9

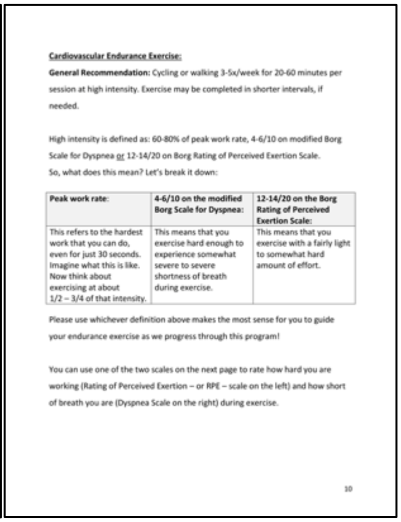

**Cardiovascular Endurance Exercise:**
**General Recommendation:** Cycling or walking 3-5x/week for 20-60 minutes per session at high intensity. Exercise may be completed in shorter intervals, if needed.

High intensity is defined as: 60-80% of peak work rate, 4-6/10 on modified Borg Scale for Dyspnea or 12-14/20 on Borg Rating of Perceived Exertion Scale.
So, what does this mean? Let's break it down:

| Peak work rate: | 4-6/10 on the modified Borg Scale for Dyspnea: | 12-14/20 on the Borg Rating of Perceived Exertion Scale: |
|---|---|---|
| This refers to the hardest work that you can do, even for just 30 seconds. Imagine what this is like. Now think about exercising at about 1/2 – 3/4 of that intensity. | This means that you exercise hard enough to experience somewhat severe to severe shortness of breath during exercise. | This means that you exercise with a fairly light to somewhat hard amount of effort. |

Please use whichever definition above makes the most sense for you to guide your endurance exercise as we progress through this program!

You can use one of the two scales on the next page to rate how hard you are working (Rating of Perceived Exertion – or RPE – scale on the left) and how short of breath you are (Dyspnea Scale on the right) during exercise.

10

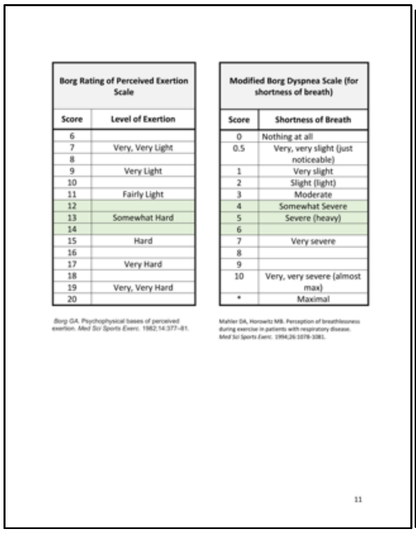

| Borg Rating of Perceived Exertion Scale | |
|---|---|
| Score | Level of Exertion |
| 6 | |
| 7 | Very, Very Light |
| 8 | |
| 9 | Very Light |
| 10 | |
| 11 | Fairly Light |
| 12 | |
| 13 | Somewhat Hard |
| 14 | |
| 15 | Hard |
| 16 | |
| 17 | Very Hard |
| 18 | |
| 19 | Very, Very Hard |
| 20 | |

Borg GA. Psychophysical bases of perceived exertion. Med Sci Sports Exerc. 1982;14:377–81.

| Modified Borg Dyspnea Scale (for shortness of breath) | |
|---|---|
| Score | Shortness of Breath |
| 0 | Nothing at all |
| 0.5 | Very, very slight (just noticeable) |
| 1 | Very slight |
| 2 | Slight (light) |
| 3 | Moderate |
| 4 | Somewhat Severe |
| 5 | Severe (heavy) |
| 6 | |
| 7 | Very severe |
| 8 | |
| 9 | |
| 10 | Very, very severe (almost max) |
| * | Maximal |

Mahler DA, Horowitz MB. Perception of breathlessness during exercise in patients with respiratory disease. Med Sci Sports Exerc. 1994;26:1078-1081.

11

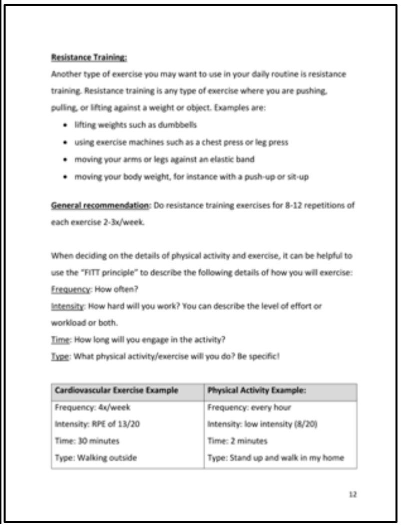

**Resistance Training:**
Another type of exercise you may want to use in your daily routine is resistance training. Resistance training is any type of exercise where you are pushing, pulling, or lifting against a weight or object. Examples are:
- lifting weights such as dumbbells
- using exercise machines such as a chest press or leg press
- moving your arms or legs against an elastic band
- moving your body weight, for instance with a push-up or sit-up

General recommendation: Do resistance training exercises for 8-12 repetitions of each exercise 2-3x/week.

When deciding on the details of physical activity and exercise, it can be helpful to use the "FITT principle" to describe the following details of how you will exercise:
Frequency: How often?
Intensity: How hard will you work? You can describe the level of effort or workload or both.
Time: How long will you engage in the activity?
Type: What physical activity/exercise will you do? Be specific!

| Cardiovascular Exercise Example | Physical Activity Example: |
|---|---|
| Frequency: 4x/week<br>Intensity: RPE of 13/20<br>Time: 30 minutes<br>Type: Walking outside | Frequency: every hour<br>Intensity: low intensity (8/20)<br>Time: 2 minutes<br>Type: Stand up and walk in my home |

12

**<u>Section 2: Physical Activity Planning, Workbook page 13</u>**
(Suggested Time: 25-30 minutes)
<u>HAPA elements addressed</u>: Action Planning, Task and Maintenance Self-Efficacy, Action, Action Control
<u>Behavior Change Techniques included</u>: Goal setting (behavior), Action Planning

The objective of this section is to develop a plan for doing one thing to increase physical activity or exercise in the next week. Allow several minutes for participants to write down their Physical Activity Action Plan for the next week. Then have some or all participants share their plan as they are willing to do so. Provide feedback about the specificity of their plan, encouraging them to identify a specific frequency, intensity, time, and type of physical activity or exercise. However, allow space for participants to determine their own plan within safe boundaries, rather than telling them what their plan should be.

If participants have difficulty generating ideas about their physical activity plan, the exercise that they have done in Phase II Pulmonary Rehabilitation can help to provide examples, e.g., for type of exercise or how to determine appropriate intensity.

Once all participants have set a plan for increased physical activity and/or exercise, have them rate how confident that are that they will be able to complete this plan and discuss as a group the ideas that participants have for how they can maximize their confidence/self-efficacy. If it helps to facilitate positive discussion, you can discuss with participants, "Why did you rate your confidence at a *(insert participant's rating)* rather than a 0?"

At the close of this session, summarize everyone's physical activity plans for the next week and highlight the positive factors related to their self-efficacy.

<u>Page 13</u>

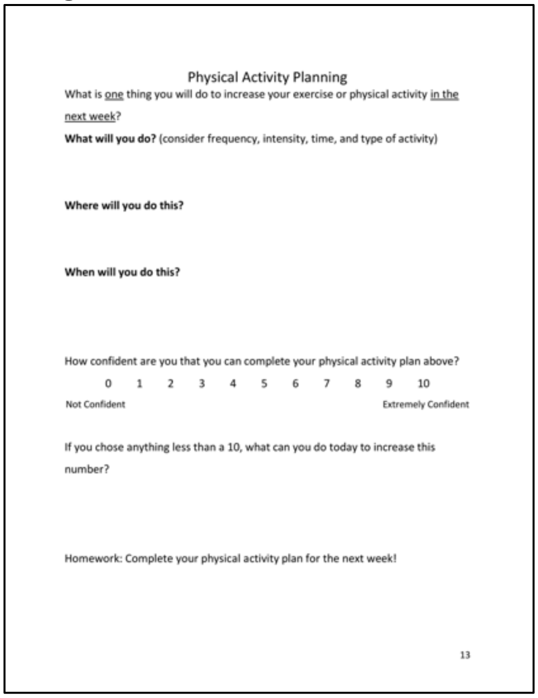

Physical Activity Planning

What is <u>one</u> thing you will do to increase your exercise or physical activity <u>in the next week</u>?

**What will you do?** (consider frequency, intensity, time, and type of activity)

**Where will you do this?**

**When will you do this?**

How confident are you that you can complete your physical activity plan above?

| 0 | 1 | 2 | 3 | 4 | 5 | 6 | 7 | 8 | 9 | 10 |
|---|---|---|---|---|---|---|---|---|---|---|
| Not Confident | | | | | | | | | | Extremely Confident |

If you chose anything less than a 10, what can you do today to increase this number?

Homework: Complete your physical activity plan for the next week!

13

# Session 3

Session 3 is divided into 4 sections: 1) Goal Setting with SMART Goals, 2) Making a Plan, 3) Identifying Resources, 4) Self-Efficacy.

**Section I: Goal Setting with SMART Goals, Workbook pages 14-15, 17**
(Suggested Time: 15-20 minutes)
HAPA elements addressed: Outcome Expectancies, Action Planning
Behavior Change Techniques included: Goal Setting (behavior), Action Planning, Comparative Imagining of Future Outcomes

Part A: The objective of this section is for participants to set goals for what they want to achieve by increasing their physical activity and/or exercise. For homework (pages 14-15), participants were assigned to read about goal setting and to write two goals using the prompts. Start by checking participants' understanding of what a SMART goal is and allow them to share the goals that they have set if they are willing to do so. Give feedback as needed to ensure that goals meet the criteria for SMART goals that are outlined in the Workbook.

Page 14

Physical Activity Planning Group Workbook
Session 3 Homework:

Your first homework is to complete your physical activity plan from Session 2! Also, before Session 3, please take a few minutes to think about your goals by reading page 14 and completing the worksheet on page 15.

Setting Goals

Setting SMART goals is a method to describe what you want to achieve. When making a change, having a target can help you:

- stay motivated
- decide on an action plan
- know when you have achieved your goal

**A SMART goal is:**

**S**pecific: What actions will you take?

**M**easurable: How will you measure whether you have met the goal?

**A**chievable: Is the goal doable?

**R**elevant: Is the result important to you?

**T**ime-bound: When do you plan to meet the goal?

*Examples*

*#1: I will walk for 30 minutes, making it at least 2 laps around the park (1/2 mile) in that time, 4 out of 7 days this week.*

*#2: I will ride a stationary bike at 10mph on level 1 resistance for 20 minutes, at least 3 out of 7 days/week, for the next month.*

*#3: I will take at least 7,000 steps per day every day for the next week.*

*#4: Every time I leave and enter my apartment without carrying anything, I will take the stairs instead of the elevator for the next month.*

14

Page 15

**Goal Setting Activity:**

Set at least two SMART goals that describe the physical activity and/or exercise that you will do. One goal should describe what you aim to do in the next week and one goal should describe what you aim to do in the next 2 weeks.

**Goal #1:** In the next week, I will:

Name each of the FITT principles included in your goal:
Frequency:

Intensity:

Time:

Type:

**Goal #2:** In the next 2 weeks, I will:

Name each of the FITT principles included in your goal:
Frequency:

Intensity:

Time:

Type:

15

Part B: Once goals are established for each group member, explore the reasons why these goals are important using the workbook prompts on page 17. Encourage the group members to examine the meaning that the improvements brought on by physical activity and exercise will hold in their lives. For instance, if they can walk longer distances and faster, they'll be able to spend more time with their spouse by going for evening walks. Or if they are more active, they'll be able to enjoy playing with their grandchildren.

Page 17

## Goal Setting – Thinking about "Why?"

After we review the goals that you set in the homework together, let's take a few minutes to reflect on why we're working on increasing physical activity and exercise in the first place.

**How will your life be better if you increase your physical activity level as you describe in your goals?** Name at least one thing you might enjoy doing if you were more physically active than you are now:

Why are those things important to you?

17

**<u>Section 2: Making a Plan, Workbook page 18</u>**
(Suggested Time 15 minutes)
<u>HAPA elements addressed</u>: Action Planning
<u>Behavior Change Techniques included</u>: Goal Setting (behavior), Action Planning

The objective for this section is to develop a specific plan for what they will do for physical activity or exercise. They've already set their goal using the FITT principle. Their plan should outline the details of how it will go. An example is included in the workbook for guidance.

<u>Page 18</u>

<u>Making a Plan</u>

What is your plan for increasing your physical activity or exercise <u>in the next two weeks</u>?

**What will you do?** (refer to your goals on p. 15!)

*Example: I will walk for 30 minutes, making it at least 2 laps around the park (1/2 mile) in that time, 4 out of 7 days this week.*

**Where will you do this?**

*Example: In the park near my house*

**When will you do this?**

*Example: On Monday, Wednesday, Friday, and Saturday mornings*

18

**<u>Section 3: Identifying Resources, Workbook pages 19-20</u>**
(Suggested Time: 15-20 minutes)
<u>HAPA elements addressed</u>: Barriers, Resources
<u>Behavior Change Techniques included</u>: Self-Monitoring of Behavior (exercise logs)

The objective for this discussion is to explore the participants' specific facilitators to physical activity.

It may be helpful for people to have examples and structure to help generate their own list of resources. Therefore, examples from the research literature are included in the Workbook. The group leader should also have a prepared list of available resources and exercise options available, if needed in the event that any participants have difficulty generating safe and effective ideas for physical activity and exercise. Resources include general resources (such as exercise logs) and community-specific resources that account for variable access to financial support, social support, and transportation. Community-specific resource examples include: local park district activities, walking groups, local gyms, local community centers, nearby parks/walking trails, discount sporting good stores/online options, and the local bus system.

**Appendix B** includes general resources such as exercise logs and physical activity/exercise instructional resources. Consider having paper copies and electronic copies available.
**Appendix C** includes an example set of community-specific resources for residents of Chicago living with chronic pulmonary disease.

**References for barriers and facilitators:**


1. Ostergaard EB, Sritharan SS, Kristiansen AD, Thomasen PM, Lokke A. Barriers and motivational factors towards physical activity in daily life living with COPD – an interview based pilot study. *Eur Clin Resp J.* 2018;5:1484654.
2. Robinson H, Williams V, Curtis F, et al. Facilitators and barriers to physical activity following pulmonary rehabilitation in COPD: a systematic review of qualitative studies. *Prim Care Resp Med.* 2018;28:19.
3. Kostell MC, Heneghan NR, Roskell C, et al. Barriers and enablers of physical activity engagement for patients with COPD in primary care. *Int J Chron Obstruct Pulmon Dis.* 2017;12:1019-1031.


<u>Page 19</u>

Resources

Now let's list the resources that you have to help you be more active. Resources can be physical, interpersonal, or intrapersonal.

<u>Physical Resources</u>:
Physical Resources are things you have around you that can help you increase your physical activity level. Examples of physical resources include:

- exercise equipment
- a walking path near your home
- a community center that offers exercise classes that you enjoy
- music to listen to while you are active
- a smart phone that will help you track exercise or steps per day
- a diary or calendar to keep track of your steps or exercise

<u>Interpersonal Resources</u>:
Interpersonal resources are other people who can help you increase your physical activity level. Examples of interpersonal resources include:

- exercise partner(s)
- supportive family members or friends
- members of this group

19

<u>Page 20</u>

<u>Intrapersonal Resources</u>:
Intrapersonal resources are characteristics within you that can help you increase your physical activity level. Examples include:

- experience with physical activity (for example, past success with exercise or sports)
- knowledge about physical activity (for example, your training in Phase II Pulmonary Rehab will help you know what to do)
- persistence
- courage
- creativity

What resources do you have that will help you increase your physical activity and exercise?

20

Additional File #3

**Section 4: Self-Efficacy, Workbook page 21**
(Suggested Time: 10 minutes)
HAPA elements addressed: Task and Maintenance Self-Efficacy
Behavior Change Techniques included: See list below

The objective of this section is to boost participants' self-confidence in their ability to increase their physical activity level.

Introduce the concept of self-efficacy and ask the participants to rate how confident they are that they can continue increase their physical activity/exercise outside of Pulmonary Rehab sessions in the next two weeks (Workbook page 21). Then, discuss with the entire group. Summarize participants' responses, reframing for generalizability when appropriate. For example, if someone answers that they would need to continue with Phase II Pulmonary Rehab long-term in order to feel confident that they could continue with exercise, re-frame as "it sounds like a group setting with a knowledgeable leader would help you increase your confidence." Then confirm whether they agree with your summary.

As you discuss what it would take to increase participants' confidence that they will be able to continue with exercise, it may be helpful to provide some example Behavior Change Techniques to consider if the participants are having difficulty generating ideas. Examples include:

- Social Comparison (e.g., provide an example of another person in a similar situation who was successful at increasing physical activity level)
- Focusing on past success (e.g., talk about times in the past where the person was successful at performing physical activity or exercise)
- Verbal persuasion about capability (e.g., tell the person that they can successfully increase physical activity, provide rationale)
- Social reward (e.g., have a friend or family member congratulate the person when they successfully complete physical activity)
- Self-monitoring of behavior (e.g., feedback about number of steps/day with a pedometer)
- Problem-solving (e.g., developing solutions that will allow the participant to avoid or overcome a barrier to successful physical activity)

An additional follow-up question that may facilitate positive discussion about self-efficacy 0-10 ratings throughout this session is "Why is your rating a *(insert participant's rating)* rather than a 0?" This question can help participants highlight the positive aspects of their confidence.

Page 21

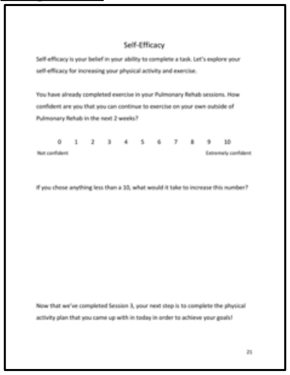
Self-Efficacy

Self-efficacy is your belief in your ability to complete a task. Let's explore your self-efficacy for increasing your physical activity and exercise.

You have already completed exercise in your Pulmonary Rehab sessions. How confident are you that you can continue to exercise on your own outside of Pulmonary Rehab in the next 2 weeks?

0 1 2 3 4 5 6 7 8 9 10
Not confident Extremely confident

If you chose anything less than a 10, what would it take to increase this number?

Now that we've completed Session 3, your next step is to complete the physical activity plan that you came up with in today in order to achieve your goals!

21

# Session 4

This session is divided into five sections: 1) Discuss Progress, 2) Identify Challenges, 3) Update Goals and Action Plan, and 4) Self-Efficacy, 5) Coping Planning

**Section 1: Discuss Progress, Workbook page 22 and 25**
(Suggested Time: 5 minutes)
HAPA elements addressed: Barriers, Coping Plans, Resources
Behavior Change Techniques included: Problem Solving, Coping Planning, Review Behavior Goals, Discrepancy Between Current Behavior and Goal

The objective for this section is for participants to evaluate their Physical Activity Action Plan implementation since the last session (2 weeks ago). Participants should have described their success with their plan in Workbook page 22 for homework. In the session they can then share with the group as they are willing. Encourage open and honest communication on level of physical activity achieved in order to maximize effectiveness of group problem-solving.

Page 22

Physical Activity Planning Group Workbook
Session 4 Homework:

Your main homework is to complete your physical activity plan!

In the day before Session 4, also complete the questions on pages 22-23 below:

What physical activity did you actually do in the last two weeks? How does what you actually did compare to your plan on p. 18?

If you were successful in enacting your plan from Session 3, Congratulations!

If you didn't achieve some of your plan, why not? And how could you adapt your plans in the future?

22

Additional File #3

**<u>Section 2: Identify Challenges, Workbook page 25</u>**
(Suggested Time: 10-15 minutes)
<u>HAPA elements addressed</u>: Barriers, Coping Plans, Resources
<u>Behavior Change Techniques included</u>: Problem Solving, Coping Planning. See additional behavior change techniques below.

The objective for this section is for participants to identify any challenges that they faced as they began implementing their exercise plan and what resources were or would be helpful. In the discussion about challenges and barriers, the focus should be on challenges/barriers that the participants actually encountered.

While strategies to overcome challenges will be most likely to succeed if the participants identify the coping strategies themselves, it may be helpful to have example strategies available. Therefore, some common barriers to physical activity and exercise for people with pulmonary disease are listed below with example strategies to overcome these challenges:

- Not knowing what to do
    - Instruction on how to perform the behavior, including skills training (review education and practice provided in Phase II Pulmonary Rehabilitation)
    - verbal persuasion about capability
    - Focus on past success
- Symptoms such as breathlessness and feeling tired
    - Information about health consequences, e.g., education about breathlessness and fatigue during exercise
    - Framing/reframing meaning of symptoms during physical activity
    - Graded tasks
- Emotions such as anxiety, fear, frustration, or embarrassment
    - monitoring of emotional consequences
    - information about emotional consequences
        - Focus on past success
        - Framing/reframing of physical activity experiences and negative emotions
        - Identity associated with changed behavior
        - Mental rehearsal of successful performance
        - Monitoring of emotional consequences of physical activity
    - Information about health consequences, e.g., education about breathlessness and fatigue during exercise
    - Framing/reframing meaning of symptoms during physical activity
    - Graded tasks
    - Reduce negative emotions, e.g., with stress management
- Lack of exercise equipment
    - Identify accessible equipment
    - Identify exercise or activity that does not require equipment
- Lack of motivation
    - Action planning for physical activity
    - Goal setting for behavior and for outcome (which is already a structured part of this intervention). Consider including functional walking tests to self-monitor improvement over time

  - Self-monitoring of behavior/consistency with exercise, e.g., exercise log
  - Self-monitoring of outcomes of behavior
  - prompts/cues to exercise
  - Comparative imagining of future outcomes
  - Self-incentive with reward for behavior
  - Social reward
- Lack of time
  - Restructure environment or task to reduce time commitment (e.g., exercise in home rather than in community, develop an efficient exercise prescription in terms of preparation or duration)
  - Habit formation
- Limited opportunities to exercise
  - Identify exercise groups or locations for physical activity/exercise
- Limited support from friends, family, or other people
  - Social support (practical): Identify an exercise partner
  - Social support (unspecified): arrange for social support in the form of praise or encouragement for increasing physical activity

**Reference for example strategies:** Michie S, Atkins L, West R. Behavior Change Wheel/Behavior Change Technique Taxonomy. Silverback Publishing; 2014.

Page 25

Physical Activity Planning - Updates

Welcome back! Two weeks ago, you came up with a plan to increase your physical activity. We'll start this session with a discussion about how it went by reviewing the homework on p. 22.

Challenges

What challenges did you face since the last session that made physical activity more difficult or prevented you from doing it altogether?

What are some ways that you could conquer these challenges?

What resources were helpful to you as you increased your physical activity last week? *Remember that your resources can be physical (e.g., equipment, space), interpersonal (e.g., supportive friends or family), or intrapersonal (personal traits that help you succeed).*

25

**Section 3: Update Goals and Action Plan, Workbook pages 23 and 26**
(Suggested Time: 10-15 minutes)
HAPA elements addressed: Outcome Expectancies, Action Planning
Behavior Change Techniques included: Goal Setting (behavior), Action Planning

The objective of this section is for participants to adjust their goals and Action Plan for the next month. Participants should have written two goals for the next month for homework already (Workbook page 23). Review these goals and guide them through making a specific plan to achieve those following the prompts on Workbook page 26. A previous example of this specific planning is on Workbook page 18.

Page 23

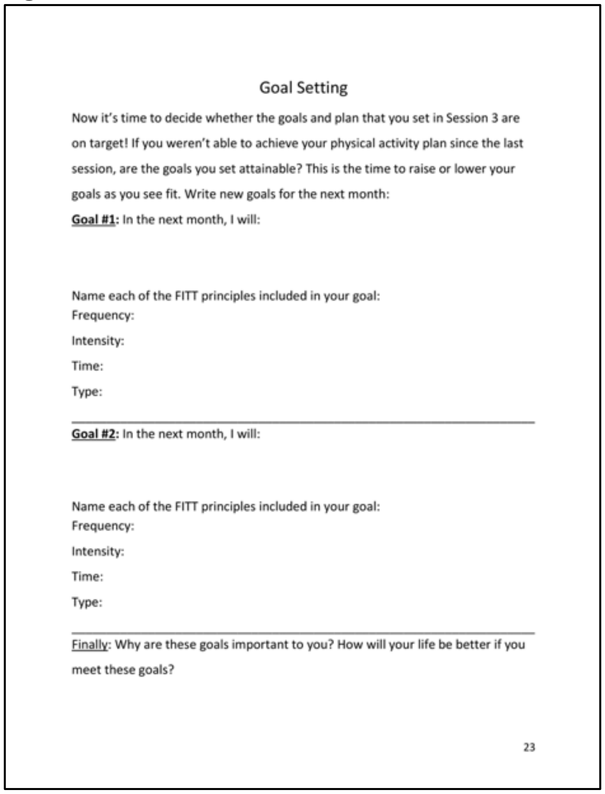

Goal Setting

Now it's time to decide whether the goals and plan that you set in Session 3 are on target! If you weren't able to achieve your physical activity plan since the last session, are the goals you set attainable? This is the time to raise or lower your goals as you see fit. Write new goals for the next month:

**Goal #1**: In the next month, I will:

Name each of the FITT principles included in your goal:
Frequency:
Intensity:
Time:
Type:

**Goal #2**: In the next month, I will:

Name each of the FITT principles included in your goal:
Frequency:
Intensity:
Time:
Type:

Finally: Why are these goals important to you? How will your life be better if you meet these goals?

23

Page 26

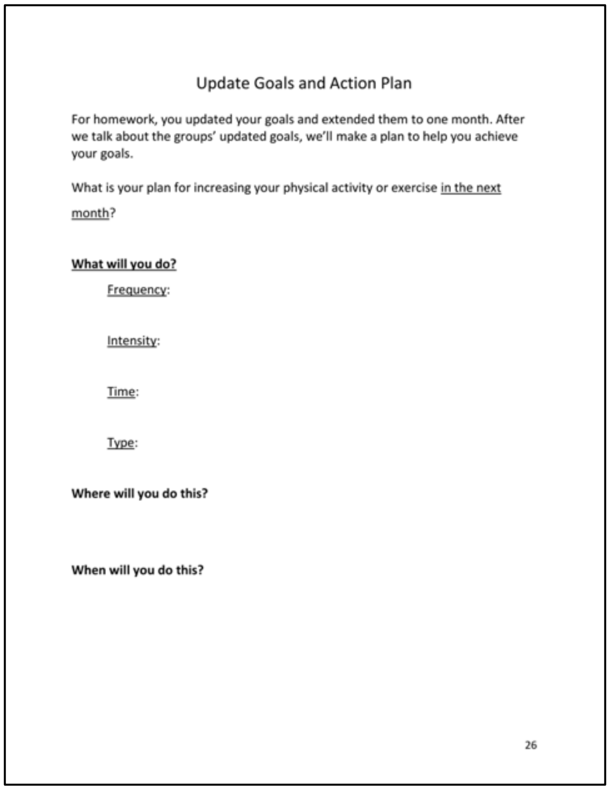

Update Goals and Action Plan

For homework, you updated your goals and extended them to one month. After we talk about the groups' updated goals, we'll make a plan to help you achieve your goals.

What is your plan for increasing your physical activity or exercise in the next month?

**What will you do?**
Frequency:
Intensity:
Time:
Type:

**Where will you do this?**

**When will you do this?**

26

Additional File #3

**Section 4: Self-Efficacy, Workbook pages 27-28**
(Suggested Time: 10 minutes)
HAPA elements addressed: Task and Maintenance Self-Efficacy
Behavior Change Techniques included: see list below

The objective of this section is to increase group members' self-efficacy for increasing physical activity and exercise.

Next, have group members answer the questions about confidence/self-efficacy on Workbook pages 27-28. Discuss as participants are willing to share and as time allows. As a reminder from Session 3, some example Behavior Change Techniques that can help increase self-efficacy include:

- Problem solving (e.g., Coping Planning, which you'll cover later in this session)
- Social Comparison
- Focus on past success
- Verbal persuasion about capability
- Social reward
- Feedback on behavior

Page 27

Self-Efficacy

It is important to maintain your increases in physical activity and exercise long-term in order to see progress. In the following two pages, we'll explore ways that you can boost your belief in your ability to keep your plan going.

How confident are you that you can keep exercising for the next month?

0 1 2 3 4 5 6 7 8 9 10

Not Confident — Extremely Confident

If you chose anything less than a 10, why is that and is there anything you can do today to increase this number?

27

Page 28

How confident are you that you can continue with this plan in the next month:

Even if you are busy, tired, or not in the mood?

0 1 2 3 4 5 6 7 8 9 10

Not Confident — Extremely Confident

Even when you cannot see positive changes right away?

0 1 2 3 4 5 6 7 8 9 10

Not Confident — Extremely Confident

Even when you face difficulties or unexpected events?

0 1 2 3 4 5 6 7 8 9 10

Not Confident — Extremely Confident

If you chose anything less than a 10 for these three questions, is there anything you can do today to increase these numbers?

28

**Section 4: Coping Planning, Workbook pages 29-30**
(Suggested Time: 20 minutes)
HAPA elements addressed: Barriers, Coping Plan, Recovery Self-Efficacy
Behavior Change Techniques included: Problem Solving, Coping Planning

The objective of this section is for participants to create a plan for how they will manage if they have a setback in their physical activity plan.

For the Activity on Workbook page 29, give participants 3-4 minutes to generate their own responses. Then give participants an opportunity to share their main challenges/barriers and strategies that they identified. In the group discussion, use a whiteboard to list challenges/barriers first – allow for participants to endorse their groupmates' stated barriers. Then brainstorm strategies together, also making a list on the whiteboard.

If time allows, role-play how the identified coping strategies will play out. For instance, for a barrier of not having enough time to exercise at the gym, coping strategies could be: 1) arranging for a spouse or partner to make dinner 3 nights per week and 2) going for a walk around the block instead of going to the gym in order to save the travel time. Coping strategy #1 may require a challenging conversation, the details of which could be role-played or discussed.

Finish this discussion by completing the ratings on Workbook page 30 and having participants use these ratings to prioritize which coping strategies will be most useful in helping to resume exercise.

Page 29

Coping Planning

You now have a solid plan for increasing your physical activity. *Excellent!*
Even with a good plan, setbacks may occur. Planning for these setbacks will ensure your success. First, we'll explore what problems are most likely to arise.
Activity:
In the left column, name up to three challenges to physical activity that you faced that are most likely to hinder your physical activity plan in the future. In the right column, describe how you can cope with each barrier or challenge and get back on track for physical activity.

| Barrier or Challenge | Coping Strategy |
|---|---|
| 1. | |
| 2. | |
| 3. | |

29

Page 30

Plan Recovery:
Imagine that you have not been exercising for a while.
How confident are you that you will be able to resume your physical activity if you miss one session of exercise or physical activity?

0 1 2 3 4 5 6 7 8 9 10
Not Confident — Extremely Confident

How confident are you that you will be able to resume your physical activity if you miss some sessions of exercise or physical activity?

0 1 2 3 4 5 6 7 8 9 10
Not Confident — Extremely Confident

Which coping strategies from the last page will be most helpful as you resume your physical activity plan?

Congratulations! You now have a complete Physical Activity Plan in place with 1) goals, 2) a specific action plan, and 3) a plan for how to recover if you stop your action plan. We'll meet again in 4 weeks to make any final changes to your action and recovery plans. We'll also talk about how to progress your physical activity plan. In the meanwhile, enjoy your more active lifestyle!

30

## Session 5

This session is divided into 4 sections: 1) Review of previous Goals, Action Plan, Coping Plan, Resource and Challenges, 2) Exercise Progression, 3) Advanced Goal Setting, and 4) Wrap-Up

**Section 1: Review of previous Goals, Action Plan, Coping Plan, Resources and Challenges, Workbook pages 31-32**
(Suggested Time: 10-15 minutes)
HAPA elements addressed: Outcome Expectancies, Action, Action Control, Barriers, Resources, Coping Plan, Recovery Self-Efficacy
Behavior Change Techniques included: Goal Setting (behavior), Action Planning, Problem Solving, Coping Planning, Discrepancy Between Current Behavior and Goal

The objective of this section is to review how things are going with participants' Physical Activity Action Plans and allow participants to make any needed adjustments to their goals, Action Plan, and Coping Plan as needed.

Start this session by reviewing the homework on Workbook pages 31-32.

As you discuss new challenges on Workbook page 32, keep in mind that the focus of discussion should generally be on the barriers to physical activity and exercise that the participants actually encountered. However, at this point, it is also a good idea to anticipate barriers that are likely to arise. An example of such a barrier is inclement weather commonly encounter in the location where participants live, such as snow and ice in a location with harsh winters.

As you discuss coping strategies on Workbook page 32, discuss interruptions in physical activity that actually happened. If participants did not have any interruptions in their Physical Activity Plan since the last session, it may be helpful to generate discussion by asking about any interruptions in physical activity or exercise that happened in the past – for instance, in Phase II Pulmonary Rehabilitation – in order to generate examples of previously successful coping strategies.

Page 31

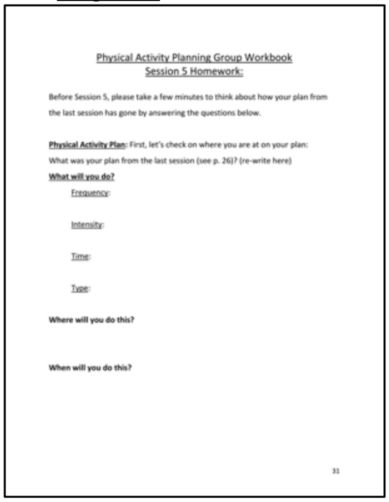
Physical Activity Planning Group Workbook
Session 5 Homework:

Before Session 5, please take a few minutes to think about how your plan from the last session has gone by answering the questions below.

**Physical Activity Plan**: First, let's check on where you are at on your plan: What was your plan from the last session (see p. 26)? (re-write here)

**What will you do?**

Frequency:

Intensity:

Time:

Type:

**Where will you do this?**

**When will you do this?**

31

Page 32

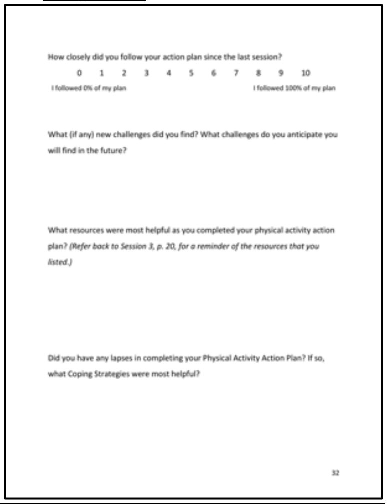
How closely did you follow your action plan since the last session?

0 1 2 3 4 5 6 7 8 9 10

I followed 0% of my plan … I followed 100% of my plan

What (if any) new challenges did you find? What challenges do you anticipate you will find in the future?

What resources were most helpful as you completed your physical activity action plan? *(Refer back to Session 3, p. 20, for a reminder of the resources that you listed.)*

Did you have any lapses in completing your Physical Activity Action Plan? If so, what Coping Strategies were most helpful?

32

**Section 2: Exercise Progression, Workbook page 34**
(Suggested Time: 15 minutes)
HAPA elements addressed: Action, Action Control
Behavior Change Techniques included: Goal Setting (behavior), Review Behavior Goals

The objective of this section is for participants to learn how to progress exercise safely and effectively. After you present the information about Exercise Progression, participants should consider how they will progress their exercise program in the future and answer the questions at the bottom of Workbook page 34. If participants are having difficulty generating answers, you can facilitate discussion in the following ways:

1) cue participants to reflect on times when they or other people in their Phase II Pulmonary Rehabilitation group progressed exercise with guidance from their care providers, or

2) provide exemplar answers for the participants to choose from. For example:

   a) You may know that it is time to progress physical activity when 1) the same exercise regimen is easier than it used to be (i.e., lower RPE rating or less dyspnea), 2) fewer breaks are needed, or 3) there is less fatigue after the bout of physical activity.

   b) You could progress your exercise in some of the following ways: 1) increase the duration of exercise with a higher number of repetitions or minutes, 2) increase the workload of exercise, e.g., with heavier weight or faster speed, 3) increase the frequency of exercise, e.g., increase from 3x/week to 4x/week, 4) complete a more difficult type of exercise, e.g., progress from the NuStep to walking or walking to running.

Page 34

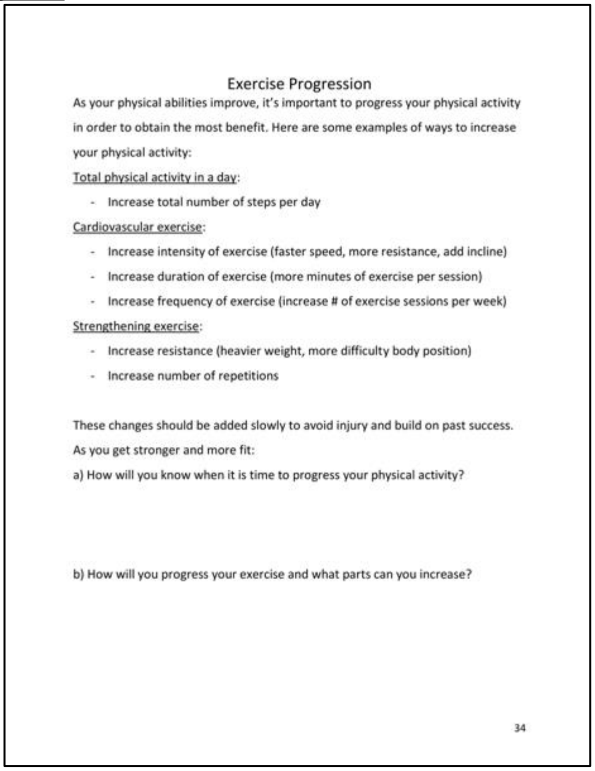

Exercise Progression

As your physical abilities improve, it's important to progress your physical activity in order to obtain the most benefit. Here are some examples of ways to increase your physical activity:

Total physical activity in a day:
- Increase total number of steps per day

Cardiovascular exercise:
- Increase intensity of exercise (faster speed, more resistance, add incline)
- Increase duration of exercise (more minutes of exercise per session)
- Increase frequency of exercise (increase # of exercise sessions per week)

Strengthening exercise:
- Increase resistance (heavier weight, more difficulty body position)
- Increase number of repetitions

These changes should be added slowly to avoid injury and build on past success. As you get stronger and more fit:

a) How will you know when it is time to progress your physical activity?

b) How will you progress your exercise and what parts can you increase?

34

Additional File #3

**Section 3: Advanced Goal Setting, Workbook page 35**
(Suggested Time: 15 minutes)
HAPA elements addressed: Action Control, Maintenance Self-Efficacy
Behavior Change Techniques included: Goal Setting (behavior), Action Planning, Review Behavior Goals

The objective for this section is for participants to develop a plan for continuing to review and advance their goals. They will also practice advancing their goals. It may be helpful to use a single participant's goal as an example for the group and advance the goal together. (This would be done as an example only and does not need to be what the participant actually does long-term.)

Page 35

## Advanced Goal Setting

Once you have achieved each goal, it is time to set a new goal. Goals should continue to be in a SMART goal format.

How will you continue to remember what your SMART goals are?

How often will you check the status of your goals and write new goals, and how will you remember to do so?

Let's practice progressing one of your goals.
Re-write one of your goals here:

When you achieve this goal, what is one way that you could write a new goal that builds on this success?

35

Additional File #3

**<u>Section 4: Wrap-Up, Workbook page 36</u>**
(Suggested Time: 10-15 minutes)

This is the group's opportunity to wrap-up. As the leader, summarize the group's accomplishments: setting goals, an action plan for physical activity, a coping plan of what to do if they have a setback, and planned for how to progress their exercise and goals for continual improvement long-term. Allow participants to comment on what components of the intervention and experience have felt the most meaningful or helpful to them and what they think will come of their new action plan.

This is an opportunity for the group to have closure and transition to a new phase of more independent planning and implementation of increased physical activity.

<u>Page 36</u>

Congratulations! You have created a Physical Activity Action Plan that sets you up for long-term success with increasing your physical activity levels. We wish much success with plenty of improvement in your physical ability, symptoms, physical and mental health, quality of life, and overall confidence to show for it!

36

Additional File #3

**Appendix A** – Example Completed Four Square Activity exploring ambivalence to change

## Group Activity: Four Square

| | **Pros** | **Cons** |
|---|---|---|
| **Increase physical activity** | • Build endurance<br>• Move easier during day-to-day activities<br>• Lose weight<br>• Happier<br>• Increase focus and concentration<br>• Sound mind – sense of satisfaction<br>• Improved balance<br>• Improve glucose levels<br>• Lower blood pressure and heart rate | • Risk of injury<br>• Muscle aches – soreness<br>• Breathlessness causes physical and mental discomfort<br>• Worry about knowing exactly how hard to push<br>• Logistics – have to manage getting out, managing portable O2 (heavy weight, limited time)<br>• Mental stress, e.g., if O2 tank is running low |
| **Keep doing what you're doing** | • Continuing at current level will lead to improvement, just over a longer period of time | • Improvement won't happen fast enough or won't happen at all without increased physical activity<br>• Easier to give up<br>• May become complacent |

In this activity, your group will make lists of the pros and cons of increasing your physical activity level with exercise. You will also make lists of the pros and cons to <u>not</u> increasing your physical activity level.

Additional File #3

**Appendix B:** The following pages include multiple examples of exercise and physical activity logs. Participants may choose one of these logs (or create their own) for tracking their physical activity, depending on their preferences and planned activities.

Additional File #3

Weekly Exercise Log ____Dates: ___/_____/_____ through ___/_____/_____

Weekly Goal:

| Date or Day of the Week | Type of Exercise | Duration (minutes) | Intensity | Other Details |
|---|---|---|---|---|
| | | | | |
| | | | | |
| | | | | |
| | | | | |
| | | | | |
| | | | | |
| | | | | |
| | | | | |
| | | | | |
| | | | | |

Additional File #3

## Weekly Exercise Log

Dates: ___/_____/_____ through ___/_____/_____

Weekly Goal:

**Sunday**

| Type of Exercise | Description (can include FITT principles) | Duration |
|---|---|---|
| | | |
| | | |
| | | |
| | | |

**Monday**

| Type of Exercise | Description (can include FITT principles) | Duration |
|---|---|---|
| | | |
| | | |
| | | |
| | | |

**Tuesday**

| Type of Exercise | Description (can include FITT principles) | Duration |
|---|---|---|
| | | |
| | | |
| | | |
| | | |

**Wednesday**

| Type of Exercise | Description (can include FITT principles) | Duration |
|---|---|---|
| | | |
| | | |
| | | |
| | | |

**Thursday**

| Type of Exercise | Description (can include FITT principles) | Duration |
|---|---|---|
| | | |
| | | |
| | | |
| | | |

**Friday**

| Type of Exercise | Description (can include FITT principles) | Duration |
|---|---|---|
| | | |
| | | |
| | | |
| | | |

**Saturday**

| Type of Exercise | Description (can include FITT principles) | Duration |
|---|---|---|
| | | |
| | | |
| | | |
| | | |

Additional File #3

# Physical Activity Log: Week from __/__/__ to __/__/__

| | | |
|---|---|---|
| **SUNDAY** | | |
| Type of Exercise | Description (FITT Principles) | Duration |
| | | |
| | | |
| **MONDAY** | | |
| Type of Exercise | Description (FITT Principles) | Duration |
| | | |
| | | |
| **TUESDAY** | | |
| Type of Exercise | Description (FITT Principles) | Duration |
| | | |
| | | |
| **WEDNESDAY** | | |
| Type of Exercise | Description (FITT Principles) | Duration |
| | | |
| | | |
| **THURSDAY** | | |
| Type of Exercise | Description (FITT Principles) | Duration |
| | | |
| | | |
| **FRIDAY** | | |
| Type of Exercise | Description (FITT Principles) | Duration |
| | | |
| | | |
| **Saturday** | | |
| Type of Exercise | Description (FITT Principles) | Duration |
| | | |
| | | |

Additional File #3

## Physical Activity Diary

If you have been completing longer walks, counting steps, and doing other daily tasks as a form of physical activity, you may log below!

Sunday:

Daily Steps/Mileage/walking time_______________________________________________

___________________________________________________________________________

Other Physical Activity_________________________________________________________

___________________________________________________________________________

Monday:

Daily Steps/Mileage/walking time_______________________________________________

___________________________________________________________________________

Other Physical Activity_________________________________________________________

___________________________________________________________________________

Tuesday:

Daily Steps/Mileage/walking time_______________________________________________

___________________________________________________________________________

Other Physical Activity_________________________________________________________

___________________________________________________________________________

Wednesday:

Daily Steps/Mileage/walking time_______________________________________________

___________________________________________________________________________

Other Physical Activity_________________________________________________________

___________________________________________________________________________

Thursday:

Daily Steps/Mileage/walking time_______________________________________________

___________________________________________________________________________

Other Physical Activity_________________________________________________________

___________________________________________________________________________

Friday:

Daily Steps/Mileage/walking time_______________________________________________

___________________________________________________________________________

Other Physical Activity_________________________________________________________

___________________________________________________________________________

Saturday:

Daily Steps/Mileage/walking time_______________________________________________

___________________________________________________________________________

Other Physical Activity_________________________________________________________

___________________________________________________________________________

Additional File #3

# Tracking your Daily Steps

| Day | Date | # of Steps | Comments |
|---|---|---|---|
| | | | |
| | | | |
| | | | |
| | | | |
| | | | |
| | | | |
| | | | |
| | | | |
| | | | |
| | | | |
| | | | |
| | | | |
| | | | |

Additional File #3

**Appendix C**: This appendix includes an example set of community-specific resources for residents of Chicago living with chronic pulmonary disease.

## Exercise Programs in Chicago's Downtown

### Shirley Ryan AbilityLab Adaptive Sports + Fitness Center

- The Shirley Ryan AbilityLab fitness center is designed to be used by those with various physical abilities and disabilities at a low cost.
- 541 N. Fairbanks, Chicago IL 60611
- (312) 238- 5001
- One time initiation fee: $35
- Yearly maintenance fee: $55 at the beginning of the year, prorated -$5 each month after January
- https://www.sralab.org/adaptive-sports-fitness-membership

### Phase III Pulmonary Rehabilitation Programs

- Phase III pulmonary rehab programs are intended to follow Phase II pulmonary rehab, providing a safe and controlled space for people with pulmonary disease to continue their fitness and maintain their health. Typically, these programs include semi-structured exercise classes and are run by pulmonary health care professionals.
- Shirley Ryan AbilityLab:
    - Fitness Center Aerobic Studio: 355 E. Erie, Chicago IL 60611
    - Monday and Wednesday 3-4 PM, Tuesday and Thursday 12-1 PM, Wednesdays and Fridays 2-3 PM
    - Shirley Ryan AbilityLab Adaptive Sports + Fitness Center membership required
    - https://www.sralab.org/pulmonary-phase-iii or call (312) 238 – 1000
- Resurrection Medical Center
    - 7345 W. Talcott Ave, Chicago 60631
    - For more information, call (773) 792-5023
- Mercy Hospital and Medical Center
    - 2525 S. Michigan Ave – Room 1201, Chicago 60616
    - For more information, call (312) 567-7088, or (312) 567-6163
- John H. Stroger Hospital of Cook County
    - 1969 W. Ogden Ave, 2nd floor Clinic T, Chicago 60612
    - For more information, call (312) 864-2923

### Community Fitness Program: Museum of Science and Industry and UCMC

- This is a community fitness walking program that occurs at the Museum of Science and Industry in the Hyde Park area of Chicago, sponsored by University of Chicago Medical Center. There are both walking groups inside the museum and fitness classes offered.
- Walking Class Hours: MWF 7:30-9:30 AM
- Fitness Class hours: MW 8:15-9:00 AM
- To register:
    - 773-702-5600: University of Chicago Medical Center Community Relations
    - Fill out form online https://redcap.uchicago.edu/surveys/?s=XELANAR9T4

Additional File #3

**Streeterville and Loop Area Indoor Malls**

Indoor mall walking is an activity in which people can exercise by walking along long corridors at indoor malls as an alternative to walking outside in inclement weather, uneven surfaces, or if walkers feel unsafe walking in their neighborhood. Some malls, like the shops at North Bridge, are designated with mall walking programs, whereas others listed below might be malls with appropriate space to walk, but do not have designated walking programs.

- The Shops at North Bridge
  - 520 N Michigan Avenue, Chicago, IL 60611
  - (312) 327-2300
  - Mall Hours:
    - Monday-Saturday: 10 AM-7PM
    - Sunday: 11 AM – 6PM
    - All mall hours designated as walking hours by AARP
- Water Tower Place
  - 835 N Michigan Ave, Chicago, IL 60611
  - (312) 440-3580
  - Mall Hours:
    - Monday-Saturday: 8-10 am
    - Sunday 8-11 am
- Block 37
  - 108 N State St, Chicago, IL 60602
  - (312) 261-4700
  - Mall Hours:
    - Monday-Saturday: 10 AM-8PM
    - Sunday: 11 AM- 6PM
- 900 North Michigan Shops
  - 900 North Michigan Avenue, Chicago, IL 60611
  - (312) 915-3916
  - Mall Hours:
    - All days 12 PM – 6 PM

**Rush Generations**

This membership is intended for how adults to be healthy as they age. Membership is open to adults of any age and includes: free lectures and workshops on health and aging-related topics, exercise and wellness classes.

- Sign-up:
  - 1-800-757-0202
  - Rush_generations@rush.edu
  - https://www.rush.edu/rush-generations to sign up online

Additional File #3

**<u>Chicago Park District Fitness Centers</u>**

These fitness centers are located throughout the city and offer cardio and strength equipment at low prices for independent workouts. For an additional fee, some locations have fitness classes as well.

- Lake Shore Fitness Center
  - 808 N. Lake Shore Drive
  - (312) 742- 7529
  - Membership: $20 a month, $60 for 3 months, $200 for the year
- Ping Tom Fitness Center
  - 1700 S. Wentworth Avenue
  - Membership: $10 a month, $30 for 3 months, $100 for the year
- Eckhart Fitness Center
  - 1330 W. Chicago Avenue
  - Membership: $15 a month, $45 for 3 months, $150 for the year
- Union Fitness Center
  - 1501 W. Randolph Street
  - Membership: $15 a month, $45 for 3 months, $150 for the year
- Full list of Chicago locations can be found at https://www.chicagoparkdistrict.com/parks-facilities/fitness-center
  - (312) 742- 7529 for questions

**<u>Chicago Park District Field Houses</u>**

- Typically sites of recreational programming and community events, and can include gymnasiums and fitness centers.
- Lake Shore Fieldhouse
  - 808 N. Lake Shore Drive
  - open 9 AM-6 PM on weekdays
- Maggie Daley Fieldhouse
  - 337 E. Randolph Street
  - (312) 747 – 6438
  - open 9 AM-5 PM on weekdays
- Jesse White Community Center
  - 410 W Chicago Avenue
  - open 9 AM-5:30PM on weekdays
- Seward Fieldhouse
  - 375 W Elm Street
  - open 8 AM-5 PM on weekdays
- Full list of Chicago locations can be found at https://www.chicagoparkdistrict.com/parks-facilities/fieldhouses

Additional File #3

**Local YMCAs**

The YMCAs of Metro Chicago offer a wide variety of fitness opportunities, community programs, arts, summer camps, and childcare. For fitness purposes, a membership at the Y offers wellness consultations, a full gym, fitness classes, personal training, and active older adult programs.

- Lakeview YMCA
  - 3333 N. Marshfield, Chicago, IL 60657
  - (773) 248-3333
- Irving Park YMCA
  - 4251 W. Irving Park Road, Chicago, IL 60641
  - (773) 777-7500
- McCormick YMCA
  - 1834 N. Lawndale Ave, Chicago, IL 60647
  - (773) 235-2525
- Kelly Hall YMCA
  - 824 N Hamlin, Chicago, IL 60651
  - (773) 886-1220
- South Side YMCA
  - 6330 S. Stony Island Avenue, Chicago, IL 60637
  - (773) 947 - 0700
- $51-53/month for 1 adult vs. $75-87/month for a family (depending on location)
- *financial assistance may be available

**Silver Sneakers**

- Medicare fitness program for 65+
- Through certain Medicare plans, available at no cost
- A variety of online and in-person fitness classes offered
  - Circuit training, yoga, Tai Chi, water aerobics, balance, strength, and more
- Visit https://tools.silversneakers.com to see if you are eligible.
